# Development, internal testing, and external validation of an interpretable machine-learning model for predicting imaging-confirmed in-hospital postoperative deep vein thrombosis in older patients with patellar fractures

**DOI:** 10.1186/s12911-026-03616-9

**Published:** 2026-06-05

**Authors:** Wei Duan, Xing Li, Yuerong Yang, Haoran Hu, Jiakai Yang, Hongxu Zhang, Yuanzheng Wei, Ruili Jia, Yubin Long

**Affiliations:** 1 Department of Orthopedic Surgery, Chengde Medical University, Baoding First Central Hospital, Baoding, Hebei 071000 China; 2 Department of Orthopedic Surgery, Baoding First Central Hospital, Baoding, Hebei 071000 China; 3 Department of Ophthalmology, Chengde Medical University, Baoding First Central Hospital, Baoding, Hebei 071000 China; 4 Department of Nephrology, Hebei Medical University, Baoding First Central Hospital, Baoding, Hebei 071000 China

**Keywords:** Patellar fracture, Deep vein thrombosis, Machine learning, XGBoost, External validation, Caprini score, Older patients

## Abstract

**Background:**

Older patients with patellar fractures may be at increased risk of postoperative deep vein thrombosis (DVT) because of trauma, perioperative immobilization, and age-related prothrombotic susceptibility. However, risk-stratification tools tailored to this specific population remain limited. We aimed to develop, internally test, and externally validate an interpretable machine-learning model, based on routinely available early admission data, for predicting imaging-confirmed in-hospital postoperative DVT in older patients with patellar fractures.

**Methods:**

This retrospective prediction-model study included an internal cohort of 741 patients aged ≥ 65 years who underwent surgery for patellar fractures between January 2017 and December 2022. The internal cohort was randomly divided into a development set (*n* = 518) and a held-out internal test set (*n* = 223). An independent external validation cohort of 273 eligible patients from a separate tertiary hospital was used to evaluate model transportability. A three-step feature-selection pipeline combining variance inflation factor screening, LASSO regression, and recursive feature elimination with cross-validation was used to identify core predictors from routine admission variables. Seven machine-learning algorithms, including reference linear benchmark models, were developed and compared. The final model was externally validated and compared with the preoperative Caprini score as an established clinical VTE risk-assessment benchmark. Model performance was assessed using the area under the receiver operating characteristic curve (AUROC), Brier score, calibration analysis, decision-curve analysis, and bootstrap optimism correction. SHapley Additive exPlanations were used to describe the behavior of the final model.

**Results:**

In the internal cohort, the overall incidence of imaging-confirmed in-hospital postoperative DVT was 18.5% (137/741). In the external validation cohort, 38 of 273 patients developed DVT. Nine predictors were retained: albumin, platelet count, fibrinogen, sodium, body mass index, total protein, cholinesterase, low-density lipoprotein cholesterol, and hemoglobin. Among the candidate models, XGBoost achieved the highest held-out internal AUROC of 0.927 (95% CI, 0.873–0.969), which was interpreted as internal testing rather than independent validation. Bootstrap optimism correction yielded an optimism-corrected AUROC of 0.904 and an optimism-corrected Brier score of 0.089. In the external validation cohort, the XGBoost model achieved an AUROC of 0.838 (95% CI, 0.758–0.904) and a Brier score of 0.081. Compared with the preoperative Caprini score, XGBoost showed higher AUROC in both the internal test set (0.927 vs. 0.687) and the external validation cohort (0.838 vs. 0.685), with lower Brier scores in both settings. Descriptive analysis of thrombus location showed that distal DVT was the predominant classifiable subtype in both the internal and external cohorts. SHAP analysis indicated that albumin, platelet count, fibrinogen, body mass index, and sodium were among the most influential predictors, but these patterns were interpreted as model-derived associations rather than causal effects or clinical thresholds.

**Conclusion:**

We developed, internally tested, and externally validated an interpretable XGBoost model based on nine routine early admission variables for predicting imaging-confirmed in-hospital postoperative DVT in older patients with patellar fractures. The model showed higher discrimination and lower prediction error than the preoperative Caprini score in both validation settings; however, because the endpoint was an imaging-ascertained composite outcome and detailed surgical and perioperative management variables were not fully captured, residual perioperative-management confounding cannot be excluded. Therefore, the model should be considered exploratory. Clinically, a high predicted risk should be interpreted as a prompt for closer postoperative surveillance and individualized clinical review, including consideration of repeat duplex ultrasonography when appropriate, verification of thromboprophylaxis adherence, hydration optimization, and reassessment of modifiable risk factors. The model should not be used alone to intensify or extend anticoagulation, change rehabilitation protocols, alter fluid management, or determine other postoperative management decisions. Further multicenter validation, recalibration, and evaluation of clinically meaningful DVT subtypes are required before clinical implementation.

**Supplementary Information:**

The online version contains supplementary material available at 10.1186/s12911-026-03616-9.

## Introduction

Patellar fractures account for approximately 1% of all skeletal injuries, and their incidence increases markedly with age. With global population aging, individuals aged ≥ 65 years—especially women—have become an increasingly affected group [1, 2]. Unlike the high-energy mechanisms typical of younger patients, patellar fractures in older adults are more often due to low-energy trauma and exhibit features of fragility fractures, frequently accompanied by age-related declines in bone quality and neuromuscular control [3, 4]. For displaced fractures that compromise the extensor mechanism, open reduction and internal fixation (ORIF) remains standard care to restore articular congruity and extensor continuity and to support rehabilitation and functional independence [5, 6].

Despite these goals, older patients undergoing ORIF for patellar fractures may face an elevated risk of venous thromboembolism (VTE). Postoperative bracing and immobilization in extension—often required to protect fixation—can promote venous stasis. Postoperative DVT has also been reported after surgical treatment of isolated patellar fractures, highlighting the need for early risk identification and stratification to support surveillance and individualized clinical review in this population [7]. In addition, older adults commonly have a more prothrombotic milieu, including disturbances in the coagulation–fibrinolysis balance and endothelial dysfunction, which may further increase thrombotic susceptibility [8]. DVT is frequently clinically silent early yet may progress to fatal pulmonary embolism (PE) or lead to post-thrombotic syndrome (PTS) with long-term morbidity [9, 10]. However, the clinical implications of postoperative DVT depend on thrombus location and symptom status. Proximal DVT and symptomatic VTE generally have greater clinical relevance than isolated distal or asymptomatic events, whereas routine postoperative imaging may detect subclinical distal thrombosis. Therefore, prediction models based on imaging-detected DVT should clearly define the endpoint and avoid overextending their conclusions to clinically severe thrombotic events.

The Caprini risk assessment model (RAM) is widely used for venous thromboembolism risk stratification by integrating comorbidities and surgery-related factors into an additive score [11]. However, in older fracture populations, the heavy weighting of age- and trauma-related factors may lead to clustering at the high-risk end and may limit discrimination for individualized risk assessment [12]. Machine-learning (ML) approaches can model complex, potentially non-linear relationships among routinely available variables and have shown promise for thrombosis-risk prediction in fracture populations [13]. Nevertheless, most published models have focused on hip fractures, arthroplasty, or broader lower-extremity trauma cohorts [14], whereas evidence specific to older patients with patellar fractures remains limited. In addition, many existing frameworks rely primarily on static clinical-history variables and may underuse the incremental predictive information contained in routine admission laboratory tests, including coagulation and nutritional markers [15]. At the same time, ML models must be evaluated against established clinical tools, externally validated, and interpreted cautiously because model-derived feature effects do not necessarily represent causal relationships or actionable clinical thresholds. Although the thrombotic risk after patellar fracture surgery is generally considered lower than that after hip fracture or major long-bone fractures, older patients with patellar fractures represent a clinically relevant subgroup for early DVT risk stratification. Patellar fractures directly involve the knee extensor mechanism, and postoperative protection commonly requires bracing or immobilization with the knee in extension, which may reduce knee motion and early functional mobility. In older adults, this local immobilization burden occurs on the background of age-related hypercoagulability, reduced physiological reserve, comorbidity, and variable preoperative surgical delay. Therefore, the clinical priority is not to position patellar fractures as a higher-risk condition than proximal femoral or tibial fractures, but to address an evidence-sparse population in which early, individualized risk stratification remains clinically useful.

Therefore, in this retrospective prediction-model study, we aimed to develop, internally test, and externally validate an interpretable ML-based prediction model using routine early admission variables to predict imaging-confirmed in-hospital postoperative DVT in older patients undergoing surgery for patellar fractures. We evaluated model discrimination, calibration, overall prediction error, decision-curve performance, and bootstrap optimism, and we compared the final model with the preoperative Caprini score. We also performed a descriptive analysis of thrombus location to clarify the clinical composition of the imaging-confirmed DVT endpoint. Because the model was intentionally based on routine early admission variables, it was not designed to replace clinical assessment of modifiable perioperative factors such as thromboprophylaxis exposure, mobilization, immobilization, hydration, or postoperative recovery. Accordingly, the model should be interpreted as an exploratory early risk-stratification and surveillance-support tool rather than as a stand-alone treatment decision system. A high predicted risk should prompt closer clinical review, consideration of repeat ultrasonography when clinically appropriate, verification of prophylaxis adherence, hydration optimization, and reassessment of modifiable risk factors, rather than automatic intensification or extension of anticoagulation, modification of rehabilitation protocols, or other direct treatment changes.

## Materials and methods

### Study design and setting

This was a retrospective prediction-model study including an internal cohort for model development and internal testing and an independent external validation cohort for external evaluation. The study was designed to develop, internally test, and externally validate a machine-learning model for predicting imaging-confirmed in-hospital postoperative deep vein thrombosis (DVT) in patients aged ≥ 65 years with patellar fractures.

The internal cohort was derived from Baoding First Central Hospital between January 2017 and December 2022. The independent external validation cohort was derived from the Third Hospital of Hebei Medical University between January 2024 and January 2026. The study protocol for the internal cohort was approved by the Ethics Committee of Baoding First Central Hospital (approval No. 2025270), and the external validation cohort was approved by the Ethics Committee of the Third Hospital of Hebei Medical University (approval No. 2023-002-1). Given the retrospective nature of the study, the requirement for written informed consent was waived by the respective ethics committees. The study was conducted in accordance with the Declaration of Helsinki and was reported with reference to the Transparent Reporting of a multivariable prediction model for Individual Prognosis or Diagnosis (TRIPOD) and TRIPOD + AI reporting recommendations for prediction-model studies. To improve reporting transparency, we explicitly described the data sources, participant eligibility criteria, prediction time point, prediction horizon, candidate predictors, outcome ascertainment, missing-data handling, preprocessing workflow, feature-selection strategy, model-development procedures, data-leakage prevention, internal testing, external validation, calibration assessment, decision-curve analysis, model interpretability, and code availability. A completed TRIPOD + AI checklist is provided in Supplementary Table S6.

This was a prediction-modeling study aimed at developing an individualized risk prediction model rather than assessing causal relationships between exposures and outcomes. The study included model development, held-out internal testing, independent external validation, comparison with the preoperative Caprini score, and bootstrap optimism correction. The external validation cohort was not used for feature selection, model training, hyperparameter tuning, threshold selection, SMOTE, recalibration, or any other model-development procedure.

### Participants

#### Case identification and data collection

We retrospectively screened consecutive patients admitted to the Department of Orthopaedics at Baoding First Central Hospital between January 2017 and December 2022 with imaging-confirmed patellar fractures and age ≥ 65 years. These patients constituted the internal cohort.

An independent external validation cohort included 273 eligible patients aged ≥ 65 years who underwent surgery for patellar fractures at the Third Hospital of Hebei Medical University between January 2024 and January 2026. The same inclusion and exclusion criteria and the same outcome definition were applied to the external validation cohort.

#### Eligibility criteria

Inclusion criteria were: (1) age ≥ 65 years; (2) imaging-confirmed patellar fracture; (3) operative treatment, such as tension band wiring, cannulated screw fixation, or other fixation methods selected by the treating surgeon; and (4) availability of sufficient electronic medical record data for outcome ascertainment and extraction of baseline clinical variables.

Exclusion criteria were: (1) pathological fracture, such as tumor-related fracture; (2) preoperative DVT detected on admission; (3) prior history of DVT or pulmonary embolism; (4) long-term preinjury anticoagulant therapy for other indications; and (5) missing primary outcome data or absence of the core baseline records required for cohort eligibility assessment.

#### Standardized perioperative management

All procedures were performed by senior orthopaedic teams according to standardized institutional pathways. Perioperative thromboprophylaxis followed the institutional protocol during hospitalization. Unless contraindicated, patients received low-molecular-weight heparin sodium for DVT prophylaxis at a standard dose of 4250 IU once daily from admission until 12 h before surgery. Anticoagulant prophylaxis was resumed 12 h after surgery when there was no active bleeding or other contraindication and was continued until hospital discharge. If postoperative DVT was diagnosed, the regimen was adjusted to low-molecular-weight heparin sodium 4250 IU twice daily according to clinical management protocols. Intermittent pneumatic compression was also used during hospitalization as mechanical prophylaxis.

Early mobilization and rehabilitation were encouraged whenever clinically feasible, whereas knee immobilization in extension was commonly used during the early postoperative period to protect fixation and the extensor mechanism. This perioperative pathway reflected routine institutional practice during hospitalization and was described to contextualize the outcome and model interpretation. However, the model was developed using routine early admission variables rather than dynamic perioperative intervention variables. Detailed patient-level information on fixation type or construct details, anesthesia method, actual anticoagulant exposure, missed doses, individualized dose adjustment, bleeding-related interruption, timing and duration of prophylaxis, duration and strictness of immobilization, rehabilitation intensity, rehabilitation adherence, perioperative fluid management, hydration status, and postoperative clinical course was not consistently available in structured form and therefore was not included in model development. Consequently, residual confounding related to perioperative management cannot be excluded.

#### Cohort definition and data split

The internal cohort included 741 eligible patients. This cohort was split using stratified random sampling in an approximately 7:3 ratio into a development set (*n* = 518) and a held-out internal test set (*n* = 223), with the event proportion preserved (random_state = 42). The development set was used for preprocessing estimation, feature selection, hyperparameter tuning, and model training. The held-out internal test set was used once for final internal testing.

**Fig. 1:**
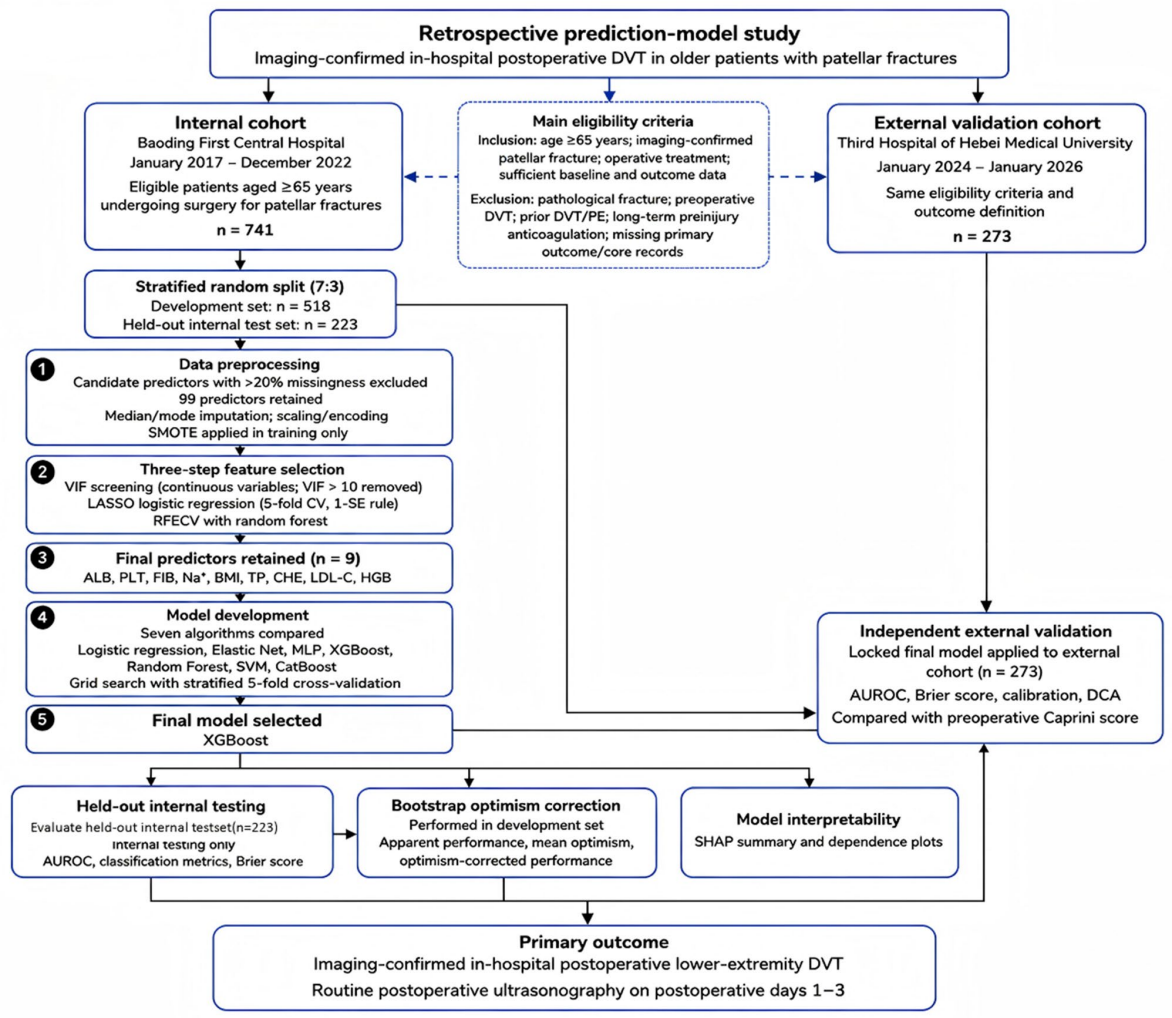
Study workflow. Flowchart showing the overall study design, cohort construction, model-development workflow, and validation strategy. The internal cohort included 741 older patients with patellar fractures from Baoding First Central Hospital and was randomly divided into a development set (*n* = 518) and a held-out internal test set (*n* = 223). The independent external validation cohort included 273 eligible patients from the Third Hospital of Hebei Medical University. The workflow included data preprocessing, three-step feature selection, model development, held-out internal testing, bootstrap optimism correction, model interpretability analysis, and independent external validation. The primary outcome was imaging-confirmed in-hospital postoperative lower-extremity DVT assessed by routine postoperative ultrasonography on postoperative days 1–3. DVT, deep vein thrombosis

The external validation cohort included 273 eligible patients from the Third Hospital of Hebei Medical University and was used only for external evaluation of the final locked model. No external-cohort data were used for model development, feature selection, hyperparameter tuning, model selection, threshold selection, SMOTE, or recalibration. The patient selection process is summarized in Fig. 1.

### Candidate predictors and data extraction

Candidate predictors were extracted from the electronic medical record system. A total of 105 initial candidate variables were considered across five domains: (1) demographics, including age, sex, body mass index (BMI), smoking history, and alcohol history; (2) comorbidities and clinical characteristics, including hypertension, diabetes mellitus, cardio-cerebrovascular disease, American Society of Anesthesiologists (ASA) class, injury mechanism, open fracture, and time from injury to surgery; (3) complete blood count and inflammatory indices, including white blood cell count, neutrophils, lymphocytes, and derived indices such as neutrophil-to-lymphocyte ratio (NLR) and systemic immune-inflammation index (SII); (4) biochemistry, including liver and renal function markers, electrolytes, and lipid parameters; and (5) coagulation parameters, including prothrombin time, fibrinogen (FIB), D-dimer, and fibrinogen-to-albumin ratio (FAR).

Only variables available at admission or during the early preoperative period were considered as candidate predictors to preserve the intended use of the model as an early in-hospital risk-stratification tool. Detailed patient-level surgical, anesthetic, and perioperative intervention variables, including fixation type or construct details, anesthesia method, exact thromboprophylaxis regimen, dose, duration, actual medication adherence, missed doses, bleeding-related interruption, duration and strictness of immobilization, rehabilitation intensity, rehabilitation adherence, perioperative fluid management, hydration status, and postoperative course, were not consistently available in structured form across the retrospective dataset and therefore were not included as candidate predictors. Accordingly, the model was not designed to estimate the causal effects of surgical approach, anesthesia method, thromboprophylaxis adequacy, immobilization severity, rehabilitation intensity, or hydration management, nor was it intended to prescribe individualized thromboprophylaxis intensity, mobilization protocols, fluid management, or other postoperative care strategies. This limitation was explicitly considered when interpreting model validity, transportability, and clinical utility.

#### Derived indices

NLR was calculated as neutrophil count divided by lymphocyte count. SII was calculated as platelet count multiplied by NLR. FAR was calculated as fibrinogen divided by albumin. All laboratory variables were taken from the first laboratory test within 24 h of admission; if multiple tests were available, the earliest value was used.

#### Baseline comparisons

Key baseline characteristics were compared between the internal cohort and the external validation cohort. Detailed baseline characteristics including all candidate predictors are provided in Supplementary Table S5. Normality of continuous variables was assessed using the Shapiro–Wilk test. Continuous variables were summarized as mean ± standard deviation or median [interquartile range], as appropriate. Group comparisons used the Student’s t test or Mann–Whitney U test. Categorical variables were summarized as number and percentage and compared using the chi-square test or Fisher’s exact test, as appropriate. A two-sided P value < 0.05 was considered statistically significant.

### Outcome definition

#### Primary outcome

The primary outcome was imaging-confirmed in-hospital postoperative lower-extremity DVT. The intended prediction time point was the early in-hospital period after admission and before postoperative outcome ascertainment, using routinely available admission and early preoperative variables. The prediction horizon extended from surgery to hospital discharge, with routine postoperative DVT screening performed on postoperative days 1–3 and additional imaging performed when clinically indicated. To ensure consistent outcome ascertainment, a standardized screening protocol was used: regardless of symptoms, all patients underwent routine bilateral lower-extremity compression ultrasonography (CUS) with color Doppler on postoperative days 1–3. For patients who developed clinical symptoms suggestive of DVT, such as limb swelling or pain, repeat ultrasonography was performed as needed. CUS was the primary diagnostic modality, and CT venography was used for confirmation when ultrasonography findings were equivocal.

#### Outcome adjudication

Outcome labels were based on formal imaging reports and discharge diagnoses according to prespecified rules: (1) outcome status was determined using all imaging examinations performed during hospitalization; (2) if multiple examinations were available, the time of the first positive imaging result was recorded as the DVT onset time; and (3) ambiguous reports were adjudicated by a senior investigator blinded to model predictions to ensure objective labeling.

#### Outcome coding and clinical interpretation

Any radiologically confirmed lower-extremity DVT detected during hospitalization was coded as an event, regardless of whether it was proximal or distal, or symptomatic or asymptomatic. Therefore, the endpoint should be interpreted as an imaging-ascertained composite in-hospital postoperative DVT outcome rather than symptomatic VTE, proximal DVT, pulmonary embolism, or a fully severity-stratified thrombotic endpoint.

#### Descriptive classification of thrombus location

DVT location was additionally classified according to available postoperative ultrasonography reports or documented venous-segment information in both the internal and external cohorts. DVT events were categorized as proximal DVT, distal DVT, mixed proximal-distal DVT, or not classifiable. Proximal DVT was defined as thrombosis involving the popliteal vein or more proximal veins, including the iliac, femoral, superficial femoral, and popliteal veins. Distal DVT was defined as thrombosis confined to below-knee veins, including the tibial, peroneal, and intermuscular veins. Mixed proximal-distal DVT was defined as simultaneous involvement of both proximal and distal venous segments. Not classifiable indicated imaging-confirmed DVT cases for which detailed venous-segment information was unavailable in the retrospective records. This classification was descriptive and was not used for model development, threshold selection, or validation.

### Data preprocessing

#### Events per variable

The development set contained 96 DVT events. The final model used nine predictors, yielding an events-per-variable value of approximately 10.7, meeting the commonly used minimum recommendation of at least 10 events per predictor parameter. The external validation cohort contained 38 DVT events and was used only for final external performance assessment.

#### Data cleaning and imputation

Cohort inclusion did not require complete availability of every candidate predictor. Instead, patients were required to have complete outcome data and sufficient baseline records for eligibility confirmation and model development. At the variable level, candidate predictors with more than 20% missingness were excluded before model building; after this step, 99 candidate predictors remained for subsequent processing.

For the remaining predictors, missing values were imputed using the median for continuous variables and the mode for categorical variables. Preprocessing was implemented using the ColumnTransformer from the scikit-learn library: (1) continuous features underwent median imputation and standardization using StandardScaler; and (2) categorical features underwent mode imputation and one-hot encoding using OneHotEncoder with handle_unknown = “ignore”. All preprocessing parameters were fitted on the development set only and then applied unchanged to the held-out internal test set and the external validation cohort.

#### Handling class imbalance

Synthetic minority oversampling technique (SMOTE) was applied during training only. To prevent data leakage, preprocessing, SMOTE, and the classifier were encapsulated in an imbalanced-learn pipeline in the order “preprocessing → SMOTE → classifier,” ensuring that SMOTE was applied only to training folds during cross-validation. Neither the held-out internal test set nor the external validation cohort was oversampled; both retained their original outcome distributions. Because SMOTE generates synthetic minority-class samples, its potential to introduce noisy synthetic observations or overfitting was considered when interpreting model performance.

### Feature selection pipeline

A three-step “funnel” feature-selection strategy was performed in the development set only. First, variance inflation factors were computed for continuous variables to assess multicollinearity; categorical variables were not included in VIF calculations but were retained for subsequent steps. Features with VIF > 10 were removed.

Second, dimensionality reduction was performed using LASSO logistic regression with L1 regularization based on LogisticRegressionCV with 5-fold cross-validation and the 1-SE rule. The 1-SE rule selected the most regularized model within one standard error of the minimum cross-validated error.

Third, recursive feature elimination with cross-validation was performed in the development set using 5-fold cross-validation and a random forest base estimator to iteratively remove less informative features and determine the optimal number and combination of predictors.

For LASSO and RFECV, predictors were transformed using the same development-set-fitted preprocessing scheme, including imputation, standardization for continuous variables, and one-hot encoding for categorical variables, to obtain a numeric design matrix. Ultimately, nine key features were retained for downstream model development: albumin, platelet count, fibrinogen, sodium, BMI, total protein, cholinesterase, low-density lipoprotein cholesterol, and hemoglobin.

After feature selection, this subset of predictors was fixed globally. The held-out internal test set and the external validation cohort were not used at any stage of feature selection. Feature selection was completed before hyperparameter tuning, model comparison, final model training, and external validation.

### Model development

Seven candidate algorithms were trained under a unified pipeline framework: conventional multivariable logistic regression, elastic-net logistic regression, multilayer perceptron, XGBoost, random forest, support vector machine, and CatBoost. Conventional logistic regression and elastic-net logistic regression were included as interpretable linear benchmark models. Probabilistic outputs were generated for all models for standardized comparison of discrimination, classification metrics, calibration, and prediction error.

#### Hyperparameter tuning

Hyperparameters were optimized using grid search with stratified 5-fold cross-validation on the development set, with mean cross-validated AUROC as the primary objective. Search spaces were defined based on common practice and model characteristics. For tree-based models, key parameters included learning rate, number of trees, and maximum depth; for support vector machine, the kernel type, regularization parameter C, and gamma where applicable were tuned. Within each cross-validation fold, preprocessing and SMOTE were refit within the training fold to avoid information leakage.

#### Final model training and selection

For each algorithm, the best hyperparameter set based on cross-validated AUROC was selected, and the model was refit on the full development set. The best-performing algorithm was selected according to overall internal performance, with emphasis on AUROC, Brier score, calibration, and decision-curve performance. After model selection, the final model was locked and then evaluated once on the held-out internal test set and once on the independent external validation cohort.

The held-out internal test set and the external validation cohort were not used for feature selection, model selection, hyperparameter tuning, threshold selection, SMOTE, or recalibration. This separation was prespecified to reduce data leakage and to align the development and validation workflow with TRIPOD + AI recommendations for prediction-model studies.

### Validation strategy and performance assessment

#### Internal testing and external validation

Model performance was evaluated on the held-out internal test set and the independent external validation cohort. The internal test set provided held-out internal performance only because it was derived from a random split of the same source cohort. It was therefore not considered independent validation. The external validation cohort, derived from a separate institution and later calendar period, was used to assess model transportability to an independent patient cohort. Uncertainty of AUROC was quantified using bootstrap resampling with 2,000 iterations and the percentile method to compute 95% confidence intervals. Bootstrap samples containing only a single class were excluded.

#### Performance metrics

Let y denote the true label and p denote the predicted probability. For a threshold t, predictions were classified as positive if p ≥ t and negative otherwise. We reported AUROC, accuracy, sensitivity, specificity, F1 score, and Brier score as the main tabulated performance metrics. Precision and Youden index were additionally calculated and visualized in the radar plot for descriptive comparison of threshold-dependent model behavior. Unless otherwise specified, a probability threshold of 0.5 was used only for standardized descriptive reporting of classification metrics across models, rather than as a clinically validated intervention threshold. Because threshold selection in a DVT prediction setting depends on the intended clinical use and the relative consequences of false-positive and false-negative classifications, discrimination, calibration, and decision-curve analysis were emphasized for model evaluation.

#### Caprini score benchmark

To assess added value over an established clinical risk-assessment tool, the final XGBoost model was compared with the preoperative Caprini score, one of the most widely used RAMs for perioperative VTE risk stratification. To maintain consistency with the intended early risk-stratification setting of the present model, the Caprini score was calculated using information available before surgery whenever possible. The Caprini score was evaluated strictly as a clinical benchmark and was not used for feature selection, SMOTE, hyperparameter tuning, model training, model selection, or recalibration. For comparison of discrimination and prediction error, AUROC and Brier score were calculated for the preoperative Caprini score in both the held-out internal test set and the external validation cohort. Threshold-dependent metrics for the Caprini score were calculated using the training-derived Youden cutoff and then applied unchanged to the internal test set and external validation cohort.

#### Discrimination and calibration

Discrimination was assessed using receiver operating characteristic curves and AUROC. Calibration was assessed using calibration plots, calibration intercept, calibration slope, and the Brier score, which together provide visual and quantitative summaries of agreement between predicted probabilities and observed outcomes. Calibration performance was evaluated in both the held-out internal test set and the external validation cohort. No post hoc recalibration was applied in the primary analysis. Therefore, external calibration results were interpreted as an assessment of model transportability rather than evidence of a recalibrated model.

#### Bootstrap optimism correction

To quantify potential optimism associated with model development and algorithm selection, bootstrap optimism correction was performed in the development set using the fixed final XGBoost model and the nine selected predictors. In each bootstrap iteration, a sample of the same size as the development set was drawn with replacement. The final model specification was trained in the bootstrap sample and evaluated both in the bootstrap sample and in the original development set. Optimism was calculated as the difference between bootstrap-sample performance and test-on-original performance. Mean optimism across bootstrap iterations was subtracted from the apparent performance to obtain optimism-corrected AUROC and Brier score. The results are reported in Supplementary Table S1.

#### Decision-curve analysis

Decision-curve analysis was performed across threshold probabilities of 0.05–0.60, using “treat-all” and “treat-none” strategies as references. Decision-curve analysis was interpreted as exploratory because the model predicts an imaging-ascertained composite DVT endpoint and does not directly specify a treatment decision threshold.

### Model interpretability

SHAP values were used to describe how individual predictors contributed to the output of the final XGBoost model. Because the final model was tree-based, TreeExplainer was used to compute SHAP values. For the nine predictors selected by the VIF–LASSO–RFECV pipeline, we generated a SHAP summary plot based on mean absolute SHAP values and SHAP dependence plots for selected variables to explore non-linear patterns learned by the model.

To aid descriptive interpretation of feature effects across value ranges, we examined approximate regions in the SHAP dependence plots where SHAP values approached or crossed zero. These regions were treated only as model-derived transition regions indicating changes in the direction of marginal contribution within the fitted model. Because such regions may depend on model structure, feature distribution, sample composition, and the external validation setting, they should not be interpreted as clinically validated cutoffs, diagnostic thresholds, biological tipping points, treatment targets, or evidence of causal effects.

### Software and reproducibility

All analyses were performed in Python version 3.9.12. Open-source libraries included scikit-learn version 1.0.2, imbalanced-learn version 0.9.0, XGBoost version 1.6.1, CatBoost version 1.0.6, SHAP version 0.41.0, statsmodels, and Matplotlib. To ensure reproducibility, a fixed random seed was used for all stochastic procedures (random_state = 42). To prevent data leakage and reduce manual variability, all steps, including preprocessing, oversampling, cross-validation, and model fitting, were implemented using pipelines.

The code used for data preprocessing, feature selection, model development, validation, calibration assessment, decision-curve analysis, and SHAP interpretation is available from the corresponding author upon reasonable request. Because the study used retrospective patient-level clinical data, the raw dataset is not publicly available due to privacy and institutional restrictions.

## Results

### Baseline characteristics of the internal and external validation cohorts

A total of 741 older patients with imaging-confirmed patellar fractures were included in the internal cohort, and the overall incidence of imaging-confirmed in-hospital postoperative DVT was 18.5% (137/741) under the routine postoperative screening protocol. This incidence refers to an imaging-ascertained in-hospital postoperative DVT endpoint rather than symptomatic VTE or proximal DVT alone. The internal cohort was randomly divided into a development set (*n* = 518) and a held-out internal test set (*n* = 223). An independent external validation cohort included 273 eligible patients from the Third Hospital of Hebei Medical University, of whom 38 developed imaging-confirmed in-hospital postoperative DVT, corresponding to an incidence of 13.9% under the same outcome definition.

Key baseline characteristics of the internal and external validation cohorts are summarized in Table 1, and the full set of baseline variables is provided in Supplementary Table S5. The internal and external cohorts differed in several baseline characteristics. Compared with the internal cohort, the external validation cohort had a lower median BMI, lower prevalence of hypertension and diabetes mellitus, longer time from injury to surgery, and higher levels of albumin, platelet count, total protein, cholinesterase, and hemoglobin-related indices. The incidence of imaging-confirmed in-hospital postoperative DVT did not differ significantly between the internal and external cohorts (18.5% vs. 13.9%, *P* = 0.106). The preoperative Caprini score was also similar between cohorts (median [IQR], 10.00 [9.00, 11.00] vs. 10.00 [9.00, 11.00]; *P* = 0.707).

**Table 1:** Key baseline characteristics of the internal and external validation cohorts

Variable	Internal cohort, *n* = 741	External validation cohort, *n* = 273	*P* value
Age, years	69 [65, 76]	68 [65, 74]	0.164
Male sex, n (%)	394 (53.2)	152 (55.7)	0.523
BMI, kg/m²	24.10 [22.13, 26.27]	23.00 [19.90, 27.50]	0.011*
Preoperative Caprini score	10.00 [9.00, 11.00]	10.00 [9.00, 11.00]	0.707
Hypertension, n (%)	305 (41.2)	70 (25.6)	< 0.001*
Diabetes mellitus, n (%)	185 (25.0)	31 (11.4)	< 0.001*
Smoking history, n (%)	47 (6.3)	27 (9.9)	0.073
Time from injury to surgery, days	4.00 [2.00, 6.00]	6.00 [3.00, 10.00]	< 0.001*
ALB, g/L	39.44 [34.81, 42.30]	41.80 [38.73, 44.31]	< 0.001*
PLT, ×10⁹/L	212.80 [175.00, 264.60]	257.00 [218.15, 302.00]	< 0.001*
FIB, g/L	3.26 [2.67, 3.96]	3.31 [2.82, 3.86]	0.551
Na⁺, mmol/L	139.96 [138.00, 142.00]	140.07 [138.50, 142.00]	0.381
TP, g/L	63.70 [59.40, 68.00]	69.22 [65.53, 72.28]	< 0.001*
CHE, U/L	7.61 [6.31, 8.92]	8.10 [6.74, 9.53]	< 0.001*
LDL-C, mmol/L	2.56 [2.02, 3.08]	2.33 [1.72, 3.17]	0.003*
HGB, g/L	126.90 [114.20, 139.00]	130.00 [118.53, 143.00]	0.060
Imaging-confirmed in-hospital postoperative DVT, n (%)	137 (18.5)	38 (13.9)	0.106

Table note: Continuous variables are presented as median [interquartile range], and categorical variables are presented as n (%). P values compare the internal cohort with the external validation cohort using the Mann–Whitney U test or Student’s t test for continuous variables and the chi-square test or Fisher’s exact test for categorical variables, as appropriate. *P < 0.05. ALB, albumin; BMI, body mass index; CHE, cholinesterase; DVT, deep vein thrombosis; FIB, fibrinogen; HGB, hemoglobin; LDL-C, low-density lipoprotein cholesterol; Na⁺, sodium; PLT, platelet count; TP, total protein

The distributions of thrombus location among patients with imaging-confirmed postoperative DVT in the internal and external cohorts are summarized in Supplementary Tables S3 and S4. In the internal cohort, thrombus location could be classified in 91 of 137 DVT cases. Among classifiable cases, distal DVT was the most common subtype, accounting for 70 of 91 cases (76.9%), followed by mixed proximal-distal DVT in 19 cases (20.9%) and proximal DVT in 2 cases (2.2%). In the external validation cohort, thrombus location could be classified in 32 of 38 DVT cases. Distal DVT was also the predominant subtype, accounting for 24 of 32 classifiable cases (75.0%), followed by mixed proximal-distal DVT in 5 cases (15.6%) and proximal DVT in 3 cases (9.4%).

### Three-step feature selection pipeline

**Fig. 2:**
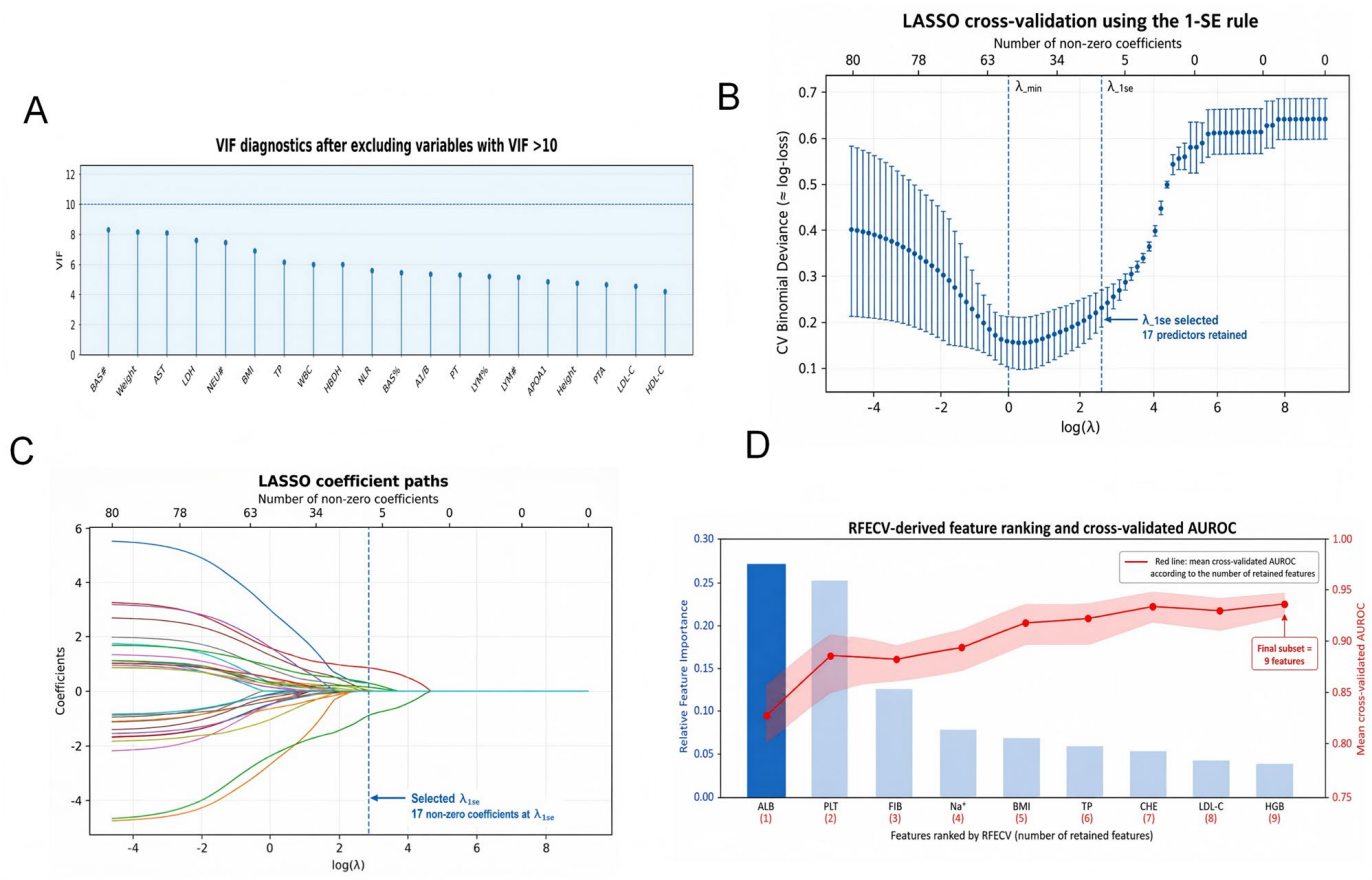
Stepwise feature-selection pipeline and results in the development set. **(A)** Variance inflation factor (VIF)-based collinearity diagnostics after exclusion of variables with VIF > 10. For clarity, the top 20 retained variables with the highest VIF values are shown. The dashed horizontal line indicates the VIF = 10 exclusion threshold. After VIF-based filtering, the feature set was reduced from 99 candidate predictors after missingness filtering to 81 predictors. **(B)** Five-fold cross-validation curve for LASSO logistic regression. The vertical dashed lines indicate λ_min and λ_1se, and the 1-SE rule was used for feature selection. At the selected λ_1se value, 17 predictors were retained. **(C)** LASSO coefficient paths across the regularization strength. The selected λ_1se value is indicated, and 17 predictors remained with non-zero coefficients at this regularization level. **(D)** RFECV results in the development set. The blue bars show the final nine retained predictors ranked by their relative contribution within the RFECV process, and the red line represents the mean cross-validated AUROC according to the number of retained features. The shaded area indicates variability across cross-validation folds. RFECV identified nine final predictors for downstream model development. AUROC, area under the receiver operating characteristic curve; LASSO, least absolute shrinkage and selection operator; RFECV, recursive feature elimination with cross-validation; VIF, variance inflation factor

To reduce redundancy and identify key predictors, we implemented a three-step “funnel” feature-selection pipeline in the development set only (Fig. 2). A total of 99 candidate variables after missingness filtering entered the pipeline. After VIF-based multicollinearity screening among continuous variables (VIF > 10; categorical variables were retained), 81 variables remained. Figure 2A shows the top 20 retained variables with the highest VIF values after exclusion of variables with VIF > 10. LASSO regression with 5-fold cross-validation and the 1-SE rule further reduced the feature set to 17 non-zero-coefficient predictors (Fig. 2B–C). Finally, RFECV using a random forest base estimator identified nine core predictors for downstream model development (Fig. 2D): albumin (ALB), platelet count (PLT), fibrinogen (FIB), sodium (Na⁺), body mass index (BMI), total protein (TP), cholinesterase (CHE), low-density lipoprotein cholesterol (LDL-C), and hemoglobin (HGB). In Fig. 2D, the red line represents the mean cross-validated AUROC according to the number of retained features, and the final RFECV-selected subset included nine predictors.

The held-out internal test set and the external validation cohort were not used during feature selection. After the nine predictors were selected, the feature set was fixed before model tuning, model comparison, internal testing, external validation, Caprini score comparison, and model interpretation.

### Internal model development and performance comparison

Based on the nine identified core features, seven machine-learning models were developed in the development set and evaluated on the held-out internal test set. These models included conventional logistic regression and elastic-net logistic regression as reference linear benchmark models. Detailed performance metrics are summarized in Table 2.

**Table 2:** Performance comparison of seven machine-learning models on the held-out internal test set (*n* = 223)

Model	AUC	95% CI	Accuracy	Sensitivity	Specificity	F1 Score	Brier
XGBoost	0.927097	0.873–0.969	0.910314	0.707317	0.956044	0.74359	0.074459
Random Forest	0.912825	0.850–0.964	0.919283	0.682927	0.972527	0.756757	0.069735
CatBoost	0.905789	0.844–0.953	0.923767	0.682927	0.978022	0.767123	0.070974
SVM	0.884749	0.807–0.947	0.901345	0.707317	0.945055	0.725	0.085626
Logistic Regression	0.855133	0.768–0.922	0.860987	0.780488	0.879121	0.673684	0.115762
Elastic Net	0.854865	0.769–0.932	0.860997	0.780488	0.879121	0.673684	0.115312
MLP	0.832887	0.746–0.909	0.816143	0.707317	0.840659	0.585859	0.157775

Note: Unless otherwise specified, threshold-dependent classification metrics, including accuracy, sensitivity, specificity, and F1 score, were calculated using a probability threshold of 0.5 on the held-out internal test set for standardized descriptive comparison across models. This threshold was not intended to represent a clinically validated decision threshold. AUROC, area under the receiver operating characteristic curve; CI, confidence interval; MLP, multilayer perceptron; SVM, support vector machine

**Fig. 3:**
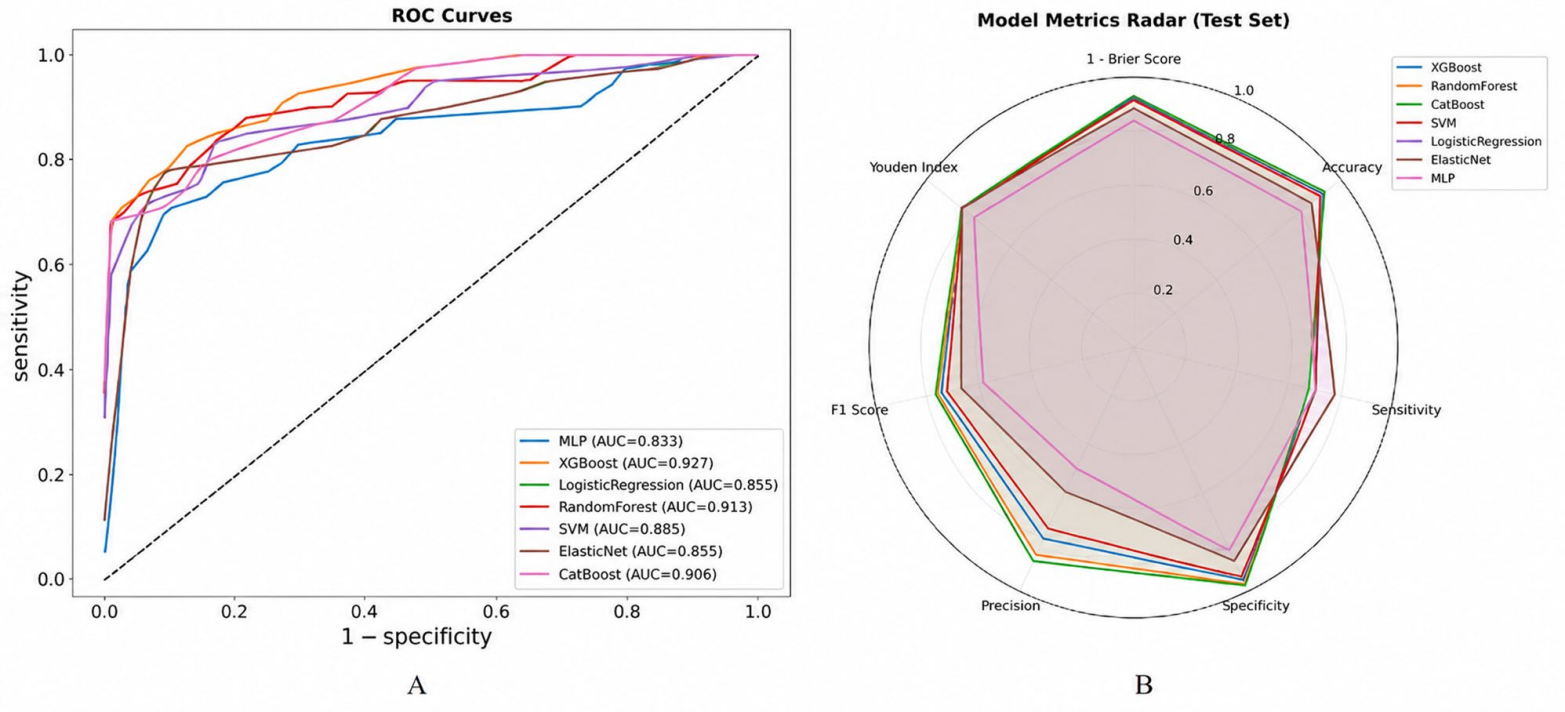
Discriminative performance and multi-metric comparison of the seven models in the held-out internal test set. (**A**) Receiver operating characteristic (ROC) curves for XGBoost, Random Forest, CatBoost, support vector machine, Logistic Regression, Elastic Net, and multilayer perceptron. (**B**) Radar plot summarizing threshold-dependent performance metrics across models, including accuracy, sensitivity, specificity, precision, F1 score, Youden index, and the transformed Brier score. The Brier score was transformed as 1 − Brier score so that higher values indicate better performance. MLP, multilayer perceptron; ROC, receiver operating characteristic; SVM, support vector machine; XGBoost, extreme gradient boosting

Among all candidate models, XGBoost achieved the highest discriminative performance in the held-out internal test set, with an AUROC of 0.927 (95% CI, 0.873–0.969) and consistently favorable ROC behavior across thresholds (Fig. 3A). Although CatBoost and Random Forest showed slightly higher accuracy and specificity, XGBoost provided a favorable trade-off between sensitivity and specificity and yielded a competitive F1 score (0.744) (Fig. 3B). Overall, tree-based ensemble methods performed better than the linear benchmark models and the other non-linear classifiers in this dataset. Random Forest (AUROC = 0.913) and CatBoost (AUROC = 0.906) were close competitors, whereas SVM showed moderate performance (AUROC = 0.885, 95% CI, 0.807–0.947). In contrast, the reference linear models, including conventional logistic regression and elastic-net logistic regression, achieved identical AUROCs of 0.855, with relatively high sensitivity but lower specificity, lower F1 scores, and higher Brier scores than XGBoost.

Regarding probabilistic prediction error, the ensemble models produced low Brier scores. Random Forest and CatBoost achieved the lowest Brier scores of 0.070 and 0.071, respectively, and XGBoost showed a similarly low Brier score of 0.074. Given its overall balance of discrimination, classification performance, calibration, and decision-curve performance, XGBoost was selected as the final model for subsequent external validation, Caprini score comparison, calibration assessment, decision-curve analysis, and interpretability analysis.

Because seven algorithms were compared and the final model was selected according to overall internal performance, potential optimism and winner’s curse were explicitly considered. To quantify potential optimism in the final model, bootstrap optimism correction was performed in the development set using the fixed final XGBoost model and the nine selected predictors. The apparent AUROC was 0.943, the mean optimism was 0.039, and the optimism-corrected AUROC was 0.904. The apparent Brier score was 0.071, the mean optimism was 0.019, and the optimism-corrected Brier score was 0.089. These results are summarized in Supplementary Table S1 and suggest that the apparent performance was optimistic to some extent.

### External validation and comparison with the preoperative Caprini score

The locked final XGBoost model was then evaluated in the independent external validation cohort from the Third Hospital of Hebei Medical University to assess model transportability across institutions. In this cohort, the model achieved an AUROC of 0.838 (95% CI, 0.758–0.904), an accuracy of 0.905, a sensitivity of 0.526, a specificity of 0.966, an F1 score of 0.606, and a Brier score of 0.081. The external ROC curve is provided in Supplementary Figure S1A. The decrease in AUROC from the held-out internal test set to the external validation cohort indicates that the internal test performance was optimistic to some extent and that external validation provides a more conservative estimate of transportability.

To evaluate whether the machine-learning pipeline provided incremental predictive value over existing clinical practice, the final XGBoost model was compared with the preoperative Caprini score as an established clinical VTE risk-assessment benchmark (Table 3). In the held-out internal test set, XGBoost achieved a higher AUROC than the Caprini score (0.927 vs. 0.687) and a lower Brier score (0.074 vs. 0.138). XGBoost also showed higher sensitivity and F1 score than the Caprini score, whereas the Caprini score showed very high specificity but low sensitivity. Table 3 provides the full comparison of discrimination, classification metrics, and prediction error between XGBoost and the preoperative Caprini score.

**Table 3:** Performance comparison between the final XGBoost model and the preoperative Caprini score

Validation cohort	Model	AUROC	95% CI	Accuracy	Sensitivity	Specificity	F1 score	Brier score
Held-out internal test set	XGBoost	0.927	0.873–0.969	0.910	0.707	0.956	0.744	0.074
	Preoperative Caprini score	0.687	0.601–0.764	0.843	0.146	1.000	0.255	0.138
External validation cohort	XGBoost	0.838	0.758–0.904	0.905	0.526	0.966	0.606	0.081
	Preoperative Caprini score	0.685	0.590–0.771	0.890	0.211	1.000	0.348	0.113

Table note: The held-out internal test set included 223 patients with 41 DVT events, and the external validation cohort included 273 patients with 38 DVT events. AUROC, area under the receiver operating characteristic curve; CI, confidence interval; DVT, deep vein thrombosis; SMOTE, Synthetic Minority Oversampling Technique; XGBoost, extreme gradient boosting. The preoperative Caprini score was evaluated as a clinical benchmark and was not used for feature selection, SMOTE, hyperparameter tuning, model training, model selection, threshold selection, or recalibration. For XGBoost, threshold-dependent metrics were calculated using a probability threshold of 0.5. For the preoperative Caprini score, threshold-dependent metrics were calculated using the training-derived Youden cutoff and then applied unchanged to the held-out internal test set and external validation cohort

In the external validation cohort, XGBoost showed higher discrimination and lower prediction error than the preoperative Caprini score, with a higher AUROC (0.838 vs. 0.685) and a lower Brier score (0.081 vs. 0.113). XGBoost showed higher sensitivity and F1 score than the Caprini score in the external cohort as well. These findings suggest that the XGBoost model provided improved discrimination and overall probability prediction error compared with the preoperative Caprini score in both validation settings.

### Model interpretability

#### Global feature importance and direction of contribution

**Fig. 4:**
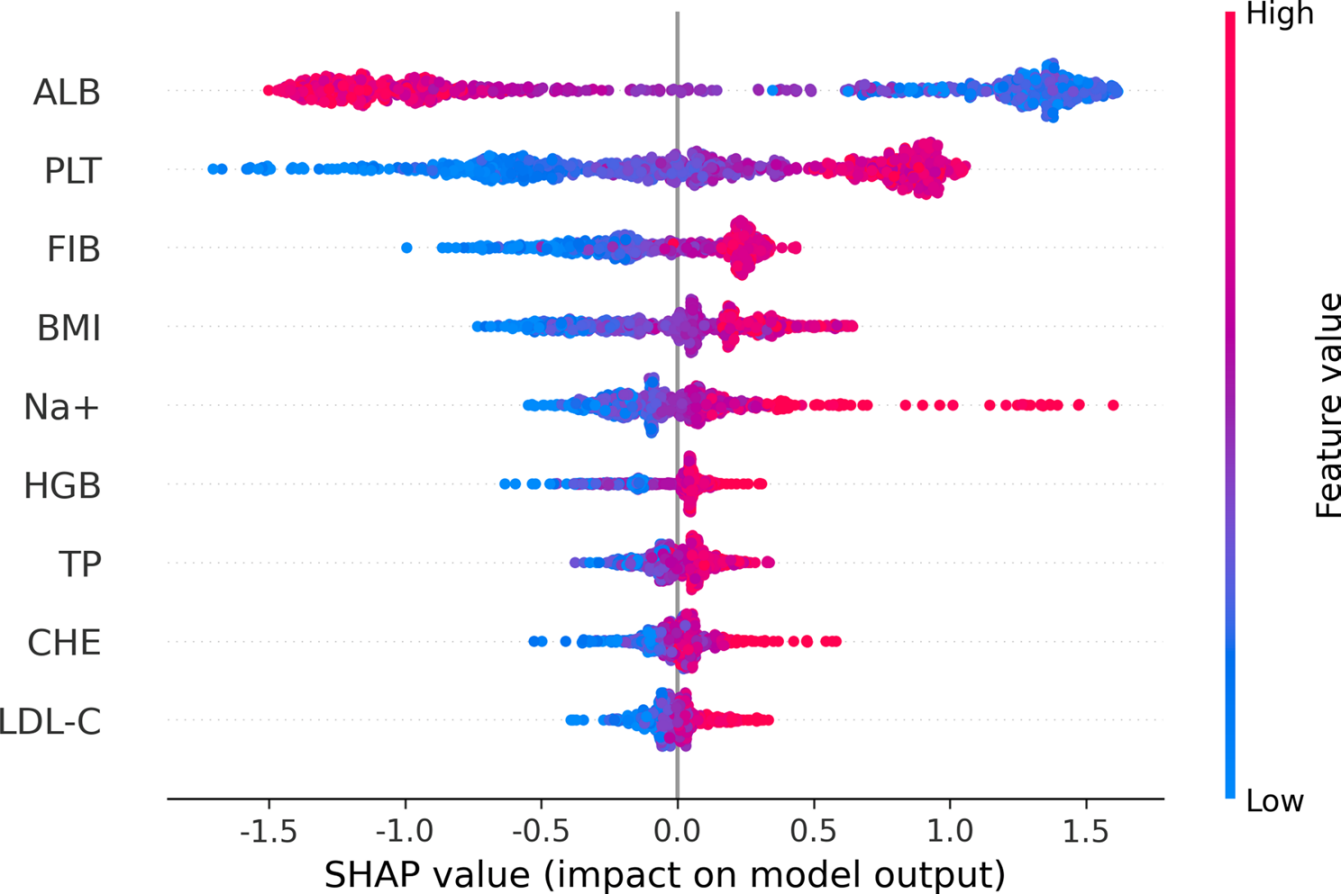
SHAP summary plot for the final XGBoost model. Each dot represents one patient. Features are ranked according to mean absolute SHAP value, reflecting global feature importance. The x-axis shows the SHAP value, which represents the impact of each feature on the model output. Positive SHAP values increase the predicted probability of imaging-confirmed in-hospital postoperative DVT, whereas negative values decrease the predicted probability. Dot color represents the original feature value, with red indicating higher values and blue indicating lower values. DVT, deep vein thrombosis; SHAP, SHapley Additive exPlanations; XGBoost, extreme gradient boosting

To describe the behavior of the final XGBoost model, SHAP values were used to quantify the contribution of each feature to the model output (Fig. 4). The SHAP summary plot ranked feature importance by mean absolute SHAP value as follows: albumin (ALB), platelet count (PLT), fibrinogen (FIB), body mass index (BMI), and sodium (Na⁺), followed by hemoglobin (HGB), total protein (TP), cholinesterase (CHE), and low-density lipoprotein cholesterol (LDL-C).

Within the fitted model, higher ALB values tended to be associated with more negative SHAP values, whereas lower ALB values tended to be associated with more positive SHAP values. In contrast, higher PLT and FIB values were more often associated with positive SHAP values and a higher predicted probability of imaging-confirmed in-hospital postoperative DVT. BMI and Na⁺ also showed overall positive contribution trends. These SHAP patterns are descriptive of the fitted model and should not be interpreted as independent causal effects, biological mechanisms, treatment targets, or clinically actionable thresholds.

#### Non-linear contribution patterns and SHAP transition regions

**Fig. 5:**
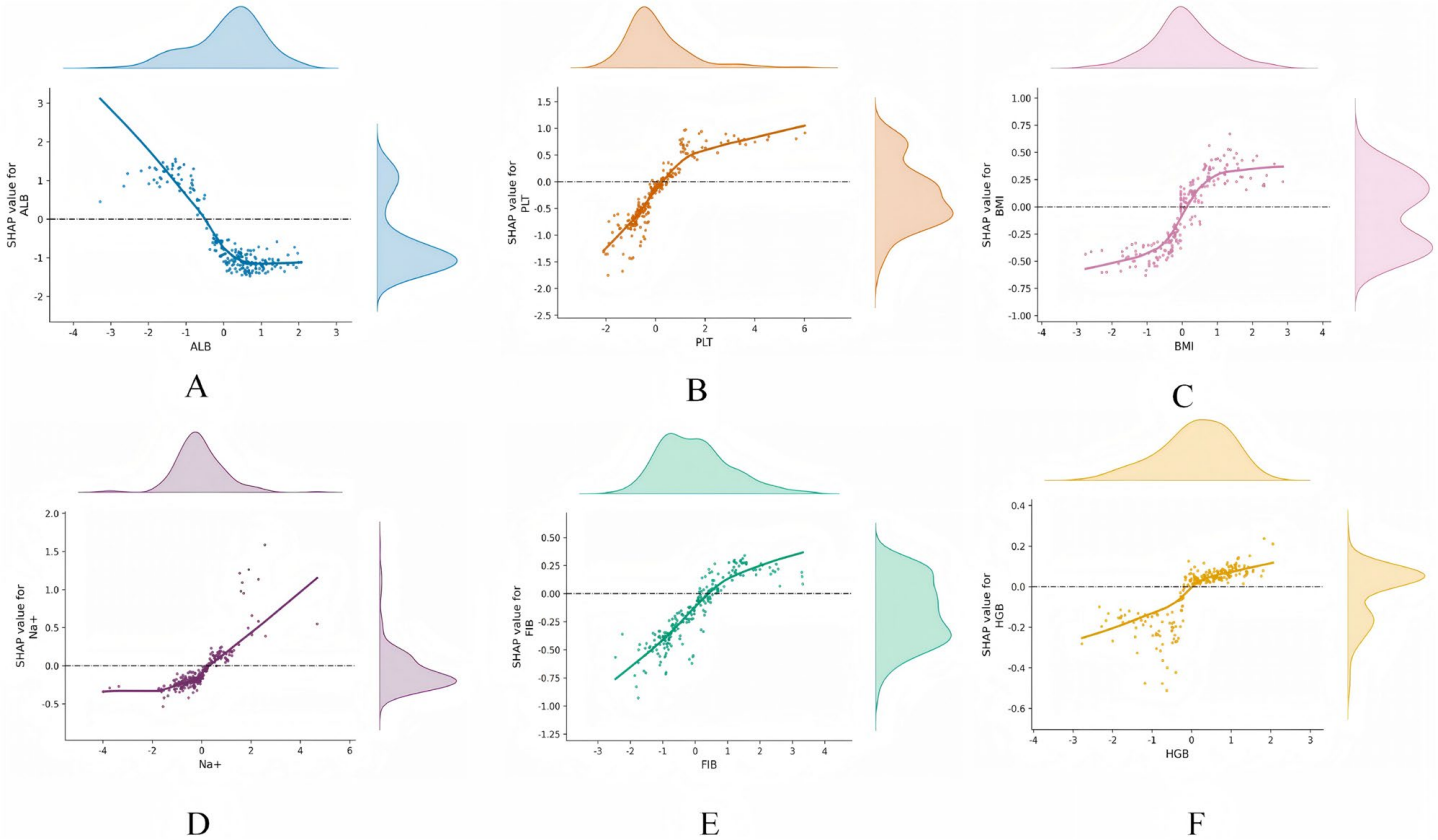
SHAP dependence plots for the top six features in the final XGBoost model. **(A**) ALB, (**B**) PLT, (**C**) BMI, (**D**) Na⁺, (**E**) FIB, and (**F**) HGB. Each dot represents one patient. The x-axis shows the standardized feature value, and the y-axis shows the corresponding SHAP value, indicating the marginal contribution of that feature to the model output. The solid curve indicates the smoothed trend, highlighting potential non-linear and plateau-like contribution patterns. The horizontal dashed line denotes SHAP = 0, where the feature contribution changes direction. Marginal distributions of feature values and SHAP values are shown for reference. These plots are descriptive of model behavior and should not be interpreted as clinically validated thresholds, biological tipping points, treatment targets, or evidence of causal effects. ALB, albumin; FIB, fibrinogen; HGB, hemoglobin; PLT, platelet count; SHAP, SHapley Additive exPlanations; XGBoost, extreme gradient boosting

SHAP dependence plots were generated for the top six features (Fig. 5A–F). Most predictors showed non-linear or plateau-like contribution patterns. ALB was associated with higher positive SHAP values at lower levels, with SHAP values decreasing rapidly and then plateauing at higher levels (Fig. 5A). SHAP values for PLT and FIB generally increased with higher values and gradually plateaued in the upper range (Fig. 5B and E). BMI exhibited a non-linear upward pattern, with SHAP values transitioning from negative to positive across an intermediate range and then stabilizing (Fig. 5C). Na⁺ showed a steeper positive increase at higher values (Fig. 5D). The contribution magnitude of HGB was smaller, but SHAP values showed a mild upward trend with increasing HGB (Fig. 5F).

To summarize these model-derived non-linear patterns, we described approximate SHAP transition regions where the dependence curves approached or crossed SHAP = 0: around 35.3 g/L for ALB, 248.4 × 10⁹/L for PLT, 3.81 g/L for FIB, 24.19 kg/m² for BMI, 141.2 mmol/L for Na⁺, and 122.5 g/L for HGB. These values are summarized in Supplementary Table S2 and are reported solely as descriptive features of the fitted model. They should not be interpreted as clinically validated decision thresholds, diagnostic cutoffs, biological tipping points, treatment targets, or evidence of causal effects.

### Calibration performance and decision-curve analysis

**Fig. 6:**
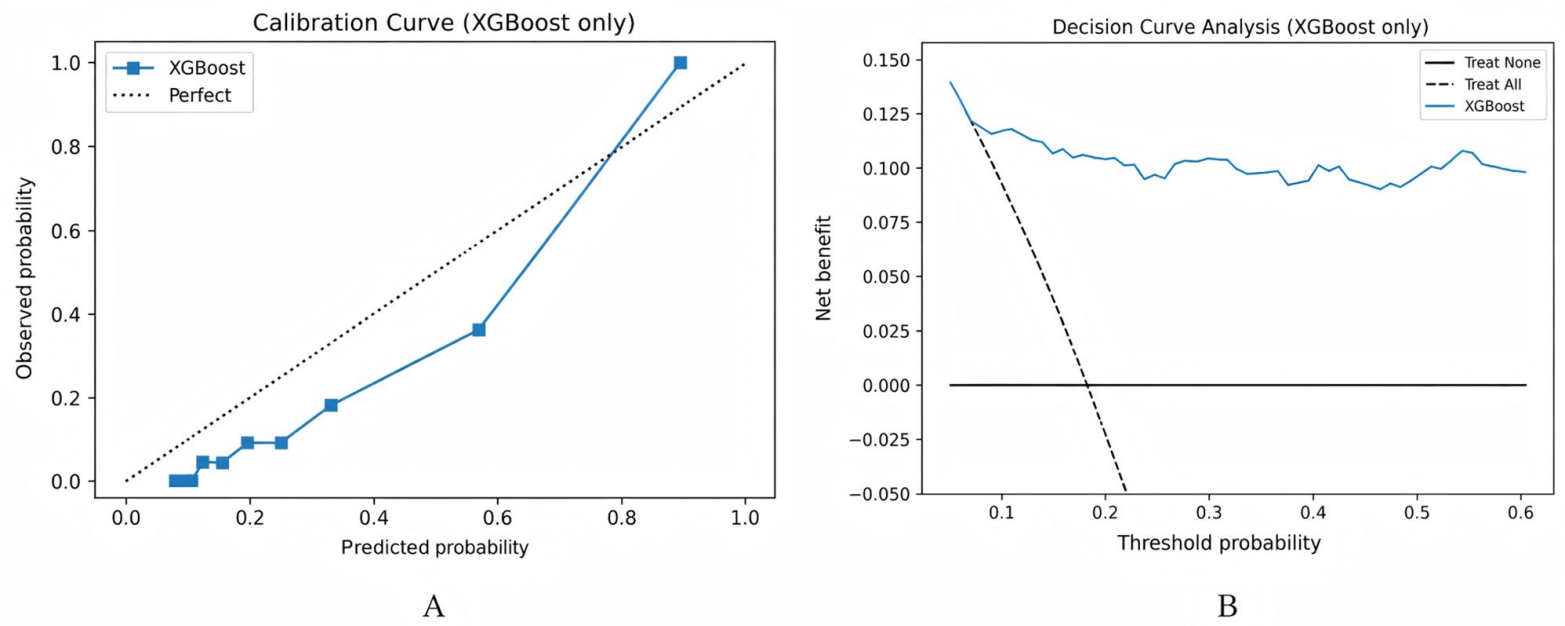
Calibration and decision-curve performance of the final XGBoost model in the held-out internal test set. (**A**) Calibration plot comparing predicted and observed probabilities of imaging-confirmed in-hospital postoperative DVT in the held-out internal test set. (**B**) Decision-curve analysis of the XGBoost model across threshold probabilities from 0.05 to 0.60, with “treat-all” and “treat-none” strategies shown as reference strategies. Decision-curve analysis was interpreted as exploratory because the model predicts an imaging-ascertained composite DVT endpoint and does not define a specific treatment threshold. DCA, decision-curve analysis; DVT, deep vein thrombosis; XGBoost, extreme gradient boosting

Calibration and decision-curve performance were evaluated for the final XGBoost model in the held-out internal test set (Fig. 6). In the held-out internal test set, the calibration curve showed that predicted probabilities were generally close to the ideal diagonal line, indicating reasonable agreement between predicted risk and observed event rates, although some deviation was present in specific probability ranges (Fig. 6A). Decision-curve analysis showed that across threshold probabilities of approximately 0.05–0.60, the XGBoost model yielded higher net benefit than the “treat-none” strategy and outperformed the “treat-all” strategy over a broad range (Fig. 6B).

In the external validation cohort, the final XGBoost model showed attenuated but acceptable discrimination and overall prediction error. External calibration analysis showed a calibration intercept of -0.484 (95% CI, -0.958 to -0.067) and a calibration slope of 0.844 (95% CI, 0.622–1.170), suggesting moderate calibration drift without post hoc recalibration. No recalibration was applied in the primary external validation analysis. The external calibration curve and decision-curve analysis are provided in Supplementary Figure S1B and S1C, respectively.

Because the model predicts an imaging-ascertained composite in-hospital postoperative DVT endpoint and does not incorporate detailed dynamic perioperative management variables, decision-curve analysis should be interpreted as exploratory rather than as evidence for a specific treatment threshold or thromboprophylaxis strategy. Taken together, the internal and external validation results suggest that the model may provide early risk-stratification information, but further multicenter validation, possible recalibration, and prospective evaluation of model-guided management pathways are required before clinical implementation.

## Discussion

In this retrospective prediction-model study with held-out internal testing and independent external validation, we developed and evaluated a machine-learning model to predict imaging-confirmed in-hospital postoperative deep vein thrombosis (DVT) in patients aged ≥ 65 years with patellar fractures. Among seven candidate algorithms, XGBoost showed high discrimination on the held-out internal test set, with an AUROC of 0.927 (95% CI, 0.873–0.969), and maintained attenuated but acceptable performance in the independent external validation cohort, with an AUROC of 0.838 (95% CI, 0.758–0.904). However, the high AUROC in the held-out internal test set should be interpreted cautiously because this test set was derived from a random split of the internal cohort and therefore does not constitute independent validation. The decrease in AUROC from the internal test set to the external validation cohort was expected and suggests that the internal estimate was optimistic to some extent, whereas the external validation provided a more conservative and clinically realistic estimate of model transportability under institutional case-mix and practice-pattern differences. The model used nine routinely available early admission variables selected using a three-step feature-selection pipeline (VIF–LASSO–RFECV) within the development set. Compared with the preoperative Caprini score, the final XGBoost model showed higher discrimination and lower prediction error in both the internal test set and the external validation cohort. Bootstrap optimism correction yielded an optimism-corrected AUROC of 0.904 and an optimism-corrected Brier score of 0.089, supporting the robustness of the final model while also indicating some degree of optimism. Calibration assessment showed reasonable internal agreement and moderate external calibration drift, suggesting that further recalibration may be needed before broader clinical use. These findings support the potential value of the model for early in-hospital risk stratification, but they should be interpreted as exploratory rather than as definitive evidence for clinical implementation.

The clinical rationale for focusing on older patients with patellar fractures requires careful interpretation. We agree that patellar fracture surgery is not generally considered to confer the same thrombotic burden as hip fracture or major long-bone trauma. However, this does not mean that DVT risk stratification is clinically irrelevant in this population. Patellar fractures impair the knee extensor mechanism, and postoperative protection frequently involves extension bracing or immobilization, which may limit early knee motion and functional mobility. In older adults, these local factors coexist with age-related prothrombotic susceptibility, comorbidity, reduced mobility, and variable surgical delay. Therefore, the purpose of the present model was not to claim that patellar fractures represent the highest-priority orthopaedic DVT population, but to provide an exploratory early risk-stratification model for an older, fracture-specific subgroup in which individualized risk assessment remains underdeveloped.

These results should be interpreted in the context of current practice and prior prediction research. Orthopaedic VTE consensus statements emphasize individualized risk assessment to guide prophylaxis, yet early and operationally actionable stratification in fracture populations remains difficult [16]. Generic risk assessment models such as the Caprini score are widely used, but their discriminative performance may be limited in orthopaedic fracture populations and may be affected by score clustering in older trauma patients [12, 17]. This has motivated the development of fracture-specific prediction tools, including perioperative DVT models for older lower-extremity fracture cohorts [18] and externally validated nomograms for older hip-fracture patients [19], while machine-learning approaches have also been explored in selected trauma populations [20, 21]. In this context, our study focuses on an evidence-sparse group—older patients with patellar fractures—and evaluates multiple candidate algorithms using only routine early admission variables. Importantly, conventional multivariable logistic regression and elastic-net logistic regression were included as internal linear benchmark models. To evaluate the model against an established clinical risk-assessment tool, we further compared the final XGBoost model with the preoperative Caprini score. XGBoost showed higher discrimination and lower prediction error than the Caprini score in both the held-out internal test set and the independent external validation cohort, with higher AUROC values and lower Brier scores. This head-to-head comparison suggests that the machine-learning pipeline may provide incremental risk-stratification information beyond the Caprini RAM in this specific population, but this finding reflects retrospective predictive performance and should not be equated with proven clinical utility. The comparison should be interpreted cautiously because this was a retrospective study, the Caprini score was not used within a standardized prospective management pathway, and the XGBoost model was not prospectively tested as part of a clinical decision-support intervention.

To contextualize the model, we summarized key SHAP patterns at the global level. These interpretability findings are descriptive and are presented to clarify how the fitted model used routine admission variables to generate predictions; they should not be interpreted as evidence of causal relationships. Within the model, higher PLT and FIB values were associated with more positive SHAP contributions, which is directionally consistent with their routine clinical relevance to coagulation status. Prior literature describes inflammation–coagulation coupling under the framework of immunothrombosis [22–24], and neutrophil extracellular trap-related scaffolding effects have also been implicated in venous thrombosis [22, 23]. BMI showed a non-linear pattern in the SHAP dependence plots, including a transition from negative to positive contribution across value ranges. This BMI-related pattern and the immobilization-related risk discussed below should be interpreted as independent clinical considerations rather than evidence of a mediation pathway. Specifically, the present model did not include structured patient-level measures of immobilization intensity, brace duration, or actual ambulation level, and no mediation analysis was performed. Therefore, our findings do not indicate that immobilization mediates the association between BMI and postoperative DVT. Instead, higher BMI may contribute to thrombotic risk through obesity-related mechanisms, whereas postoperative extension bracing and reduced mobility may contribute to venous stasis through a separate pathway. Evidence from lower-limb trauma with temporary immobilization documents increased VTE risk, and risk-stratification tools such as TRiP(cast) incorporate immobilization intensity and patient factors [25, 26]. Dose–response meta-analyses also report a positive association between increasing BMI and VTE risk [27], which is directionally consistent with, but does not validate, the pattern learned by the present model. Therefore, the observed SHAP patterns should be viewed as model-derived contribution profiles rather than mechanistic explanations, independent causal effects, validated clinical thresholds, or actionable treatment targets.

Separately from the BMI-related model pattern, a patella-fracture–specific clinical consideration is the frequent use of extension immobilization after injury or fixation. Contemporary guidance commonly recommends bracing with the knee locked in extension during early ambulation, with knee flexion advanced in a staged manner to protect the extensor mechanism and fixation [28, 29]. This care pathway can limit knee excursion and functional mobility in the early postoperative period, even when weight bearing is permitted. These pathway characteristics are relevant when interpreting stasis-related risk during hospitalization. However, immobilization intensity, brace duration, actual ambulation level, and rehabilitation adherence were not consistently available as structured patient-level variables. Therefore, the present model may partly capture indirect correlates of immobilization or perioperative management rather than the direct causal effects of these modifiable factors. This limitation is important when considering model transportability across institutions with different rehabilitation and thromboprophylaxis protocols.

The absence of detailed surgical, anesthetic, and modifiable perioperative intervention variables has important implications for clinical interpretation and potential confounding bias. Although we described the institutional thromboprophylaxis and rehabilitation pathway, the retrospective dataset did not consistently capture fixation type or construct details, anesthesia method, actual anticoagulant exposure, missed doses, individualized dose adjustment, prophylaxis duration, bleeding-related interruption, duration and strictness of immobilization, rehabilitation intensity, rehabilitation adherence, perioperative fluid management, hydration status, or postoperative recovery trajectory in structured form. These variables may directly influence postoperative DVT risk and may also differ across institutions, thereby affecting model validity and transportability. Their absence means that the model may partly capture proxies for surgical complexity, immobilization burden, prophylaxis adequacy, rehabilitation practice, or broader institutional care patterns rather than true thrombotic susceptibility alone. Therefore, the model should not be interpreted as determining whether anticoagulation should be intensified, extended, withheld, or modified for an individual patient, nor should it be used to directly change rehabilitation protocols, fluid management, or other postoperative care strategies. Rather, a high predicted risk should be treated as a clinical warning signal that prompts closer postoperative surveillance and individualized clinical review. In practical terms, this may include careful reassessment of modifiable risk factors, verification that pharmacologic and mechanical thromboprophylaxis is being delivered according to institutional protocols, identification of missed anticoagulant doses or contraindications when such information is available, optimization of hydration status, encouragement of early mobilization when clinically feasible, and consideration of repeat duplex ultrasonography when symptoms develop or when the clinician judges that additional surveillance is appropriate. Final management decisions should remain based on comprehensive clinical assessment, bleeding risk, fracture stability, rehabilitation tolerance, institutional protocols, and physician judgment. Prospective studies with structured recording of fixation method, anesthesia method, actual thromboprophylaxis exposure, missed anticoagulant doses, immobilization duration and severity, rehabilitation intensity, ambulation level, hydration status, and postoperative recovery are required. Future versions of the model should incorporate these dynamic perioperative variables and postoperative trajectories, rather than relying only on static admission and preoperative variables, to improve transportability across institutions and enhance clinical actionability. Such model-guided management pathways should then be prospectively evaluated to determine whether they improve clinically meaningful outcomes without increasing bleeding or other harms.

Beyond these signals, the model retained variables that reflect nutritional/protein reserve and internal milieu. ALB showed a stable protective direction, consistent with large-scale evidence linking hypoalbuminemia to higher VTE risk [30] and orthopaedic data associating lower ALB with higher DVT risk [31]. TP and CHE were retained as well, and both have been used as indicators of nutritional and synthetic reserve in older patients [32, 33]. Na⁺ and HGB showed overall positive trends; experimental work links higher sodium exposure to endothelial procoagulant signaling [34], and clinical literature describes dehydration and hemoconcentration as prothrombotic states [35], while prospective cohort data associate higher hematocrit/hemoglobin with higher VTE incidence [36]. LDL-C was also retained. Observational studies and genetic analyses report associations between lipid phenotypes and VTE, with heterogeneity across lipid fractions and study designs [37, 38]. Several predictors showed plateau effects and non-linear shapes in the SHAP dependence plots; the approximate SHAP transition regions describe the fitted model’s learned contribution patterns and should not be interpreted as clinical decision thresholds, diagnostic cutoffs, biologically validated cut points, treatment targets, or causal inflection points. D-dimer and inflammatory indices, such as NLR and SII, were higher in univariate comparisons but were not retained in the final feature set. This pattern is consistent with redundancy or collinearity with retained predictors and limited incremental value for early admission prediction in this dataset.

The observed DVT incidence should be interpreted in relation to the outcome definition and screening strategy. The internal incidence of 18.5% and external incidence of 13.9% refer to imaging-confirmed in-hospital postoperative DVT detected under a routine postoperative ultrasonography protocol, rather than symptomatic VTE, proximal DVT, PE, or clinically severe thrombotic events. Reported DVT incidence in patellar-fracture populations varies across studies because of differences in observation windows, screening intensity, and whether distal or intermuscular venous thrombosis is counted [39]. Therefore, the incidence observed in the present study should not be interpreted as the incidence of clinically overt or proximal VTE, but rather as the event rate for the prespecified imaging-ascertained in-hospital DVT endpoint.

Consistent with this incidence interpretation, the clinical interpretation of the outcome also requires caution. The primary endpoint was imaging-confirmed in-hospital postoperative DVT detected during hospitalization. Routine postoperative ultrasonography reduced symptom-driven detection bias, but the endpoint remains an imaging-ascertained composite outcome. In the revised analysis, we added a descriptive classification of thrombus location in both the internal and external cohorts. Among classifiable cases, distal DVT was the predominant subtype in both cohorts. This distribution is consistent with routine screening studies in orthopaedic trauma, in which subclinical distal thrombosis is commonly detected. However, detailed venous-segment information was unavailable in a proportion of cases, and symptomatic status was not consistently documented in the retrospective records. Therefore, the model should not be interpreted as predicting symptomatic VTE, proximal DVT, pulmonary embolism, or clinically severe thrombotic events. Future studies should evaluate clinically meaningful DVT subtypes separately, especially symptomatic DVT and proximal DVT, when sufficient event numbers are available.

Several aspects of model validation and clinical applicability deserve emphasis. First, the final locked model was evaluated in an independent external validation cohort derived from the Third Hospital of Hebei Medical University. This cohort was not used during feature selection, model development, hyperparameter tuning, threshold selection, SMOTE, recalibration, or any other model-development procedure. Therefore, the external validation results reflect the transportability of the locked model to an independent institutional setting rather than performance after local model updating. Second, the lower AUROC in the external cohort compared with the held-out internal test set is expected and may reflect differences in case mix, care pathways, rehabilitation practices, imaging documentation, and measurement patterns between institutions. Third, the external calibration intercept and slope suggested moderate calibration drift. Because no post hoc recalibration was applied in the primary analysis, the model should not be considered ready for direct deployment without local validation and, if needed, recalibration. Fourth, bootstrap optimism correction in the development set indicated that the apparent model performance was optimistic to some extent. Therefore, the model should be considered promising for early risk stratification but not yet ready for direct clinical deployment without further multicenter validation, prospective evaluation, and potential local recalibration.

Several limitations should be acknowledged. First, although this study included an independent external validation cohort, it remained retrospective, and selection bias, residual confounding, and differences in institutional care pathways could not be fully eliminated. The external validation cohort also contained only 38 DVT events, which limits the precision of external calibration and subgroup analyses. Second, the primary endpoint was imaging-confirmed in-hospital postoperative DVT rather than symptomatic VTE, proximal DVT, pulmonary embolism, or a severity-stratified thrombotic endpoint. Although we added descriptive thrombus-location classification, symptomatic status and detailed venous-segment information were not consistently available in all cases. Third, although perioperative care generally followed institutional pathways, key surgical, anesthetic, and modifiable patient-level intervention and management factors were not comprehensively captured in structured form, including fixation type or construct details, anesthesia method, actual thromboprophylaxis regimen, dose, duration, adherence, missed anticoagulant doses, individualized dose adjustment, bleeding-related interruption, duration and strictness of immobilization, rehabilitation intensity, rehabilitation adherence, ambulation level, perioperative fluid management, hydration status, and postoperative clinical course. This represents a major limitation because these variables are clinically relevant determinants of postoperative DVT risk and may also vary substantially across institutions, thereby influencing both model transportability and clinical actionability. Their absence means that the model may partly learn proxies for surgical complexity, anesthesia-related care patterns, prophylaxis adequacy, immobilization burden, rehabilitation practice, perioperative management, or institutional care patterns rather than true thrombotic susceptibility alone. Therefore, residual confounding and confounding by surgical and perioperative management cannot be excluded, and the model should not be used as a stand-alone tool to determine anticoagulation intensity, rehabilitation protocols, fluid management, or other postoperative management decisions. Future model iterations should incorporate dynamic perioperative variables and postoperative trajectories, including time-varying thromboprophylaxis exposure, missed-dose information, immobilization duration and strictness, rehabilitation intensity, ambulation level, hydration status, and postoperative recovery course. Incorporating these variables may improve model transportability across institutions with different care pathways and may enhance clinical actionability by distinguishing baseline thrombotic susceptibility from modifiable perioperative risk. Fourth, the feature set was intentionally limited to early admission variables to support early risk stratification, but this design excluded dynamic postoperative trajectories and adherence measures. Although we added a head-to-head comparison with the preoperative Caprini score, this comparison was retrospective and did not evaluate whether either strategy changed clinician behavior, thromboprophylaxis decisions, bleeding risk, or patient outcomes. Fifth, despite strict separation of development, held-out internal testing, and external validation, overfitting and winner’s curse remain possible because seven algorithms were compared and the number of DVT events was modest. The held-out internal test set was derived from a random split of the internal cohort and therefore should be interpreted as internal testing rather than independent validation. Although bootstrap optimism correction and independent external validation were added to quantify and test model optimism, the high internal AUROC may still overestimate performance in other settings. SMOTE was applied only within the development process and never to the held-out internal test set or external validation cohort; however, because SMOTE generates synthetic minority-class samples rather than real patients, it may create noisy or less representative DVT patterns, exaggerate minority-class separation during training, and contribute to overfitting. Finally, SHAP improved model transparency but did not establish causality, biological mechanisms, validated clinical thresholds, or treatment targets; the SHAP transition regions should therefore be considered descriptive model-behavior summaries only. Prospective studies should assess whether model-guided risk stratification improves clinically meaningful outcomes, such as symptomatic DVT, proximal DVT, pulmonary embolism, bleeding, and net clinical benefit.

## Conclusion

We developed, internally tested, and externally validated an interpretable XGBoost model based on nine routine early admission variables for predicting imaging-confirmed in-hospital postoperative DVT in older patients with patellar fractures. The model showed high held-out internal discrimination and low prediction error, but this internal performance should be interpreted cautiously because the test set was derived from a random split of the same source cohort. In the independent external validation cohort, the model maintained acceptable, although attenuated, performance. Compared with the preoperative Caprini score, the XGBoost model showed higher discrimination and lower prediction error in both validation settings. However, because the endpoint was an imaging-ascertained composite outcome rather than symptomatic VTE, proximal DVT, pulmonary embolism, or a clinically severity-stratified thrombotic endpoint, and because detailed surgical, anesthetic, and perioperative management variables were not fully captured, the model should be considered exploratory. In clinical practice, the model is best regarded as a surveillance-support tool: high predicted risk may justify closer monitoring, individualized clinical review, verification of prophylaxis adherence, hydration optimization, and consideration of repeat ultrasonography when appropriate, but should not automatically trigger intensified or extended anticoagulation, altered rehabilitation protocols, fluid-management changes, or other direct treatment changes. Further multicenter validation, local recalibration when needed, and evaluation of clinically meaningful DVT subtypes and prospective model-guided management pathways are required before clinical implementation.

## Supplementary Information

Below is the link to the electronic supplementary material.

Supplementary Material 1

Supplementary Material 2

Supplementary Material 3

Supplementary Material 4

Supplementary Material 5

Supplementary Material 6

Supplementary Material 7

## Data Availability

The datasets generated and/or analyzed during the current study are available from the corresponding author on reasonable request.

## Abbreviations

ALB: Albumin
ASA: American Society of Anesthesiologists
AUROC: Area under the receiver operating characteristic curve
BMI: Body mass index
CHE: Cholinesterase
CI: Confidence interval
CUS: Compression ultrasonography
DCA: Decision-curve analysis
DVT: Deep vein thrombosis
FAR: Fibrinogen-to-albumin ratio
FIB: Fibrinogen
HGB: Hemoglobin
LDL-C: Low-density lipoprotein cholesterol
ML: Machine learning
NLR: Neutrophil-to-lymphocyte ratio
ORIF: Open reduction and internal fixation
PE: Pulmonary embolism
PLT: Platelet count
PTS: Post-thrombotic syndrome
RAM: Risk assessment model
RFECV: Recursive feature elimination with cross-validation
SHAP: SHapley Additive exPlanations
SII: Systemic immune-inflammation index
SMOTE: Synthetic Minority Oversampling Technique
TP: Total protein
TRIPOD: Transparent Reporting of a multivariable prediction model for Individual Prognosis or Diagnosis
VIF: Variance inflation factor
VTE: Venous thromboembolism
Na⁺: Sodium
XGBoost: Extreme gradient boosting
