# Supplementary material for: Development, internal testing, and external validation of an interpretable machine-learning model for predicting imaging-confirmed in-hospital postoperative deep vein thrombosis in older patients with patellar fractures
Source: BMC Med Inform Decis Mak. 2026 Jun 5;26:309. doi: 10.1186/s12911-026-03616-9 (PMC13459360; doi:10.1186/s12911-026-03616-9)
Supplement: Supplementary file 1 [file 12911_2026_3616_MOESM2_ESM.docx]

**Supplementary Table S1. Bootstrap optimism-corrected performance of the final XGBoost model.**

| Metric | Apparent performance | Mean optimism | Optimism-corrected performance |
| --- | --- | --- | --- |
| AUROC | 0.943 | 0.039 | 0.904 |
| Brier score | 0.071 | 0.019 | 0.089 |

**Table note:** AUROC, area under the receiver operating characteristic curve; XGBoost, extreme gradient boosting. Bootstrap optimism correction was performed in the development set using the fixed final XGBoost model and the nine selected predictors.
