# Supplementary material for: Development, internal testing, and external validation of an interpretable machine-learning model for predicting imaging-confirmed in-hospital postoperative deep vein thrombosis in older patients with patellar fractures
Source: BMC Med Inform Decis Mak. 2026 Jun 5;26:309. doi: 10.1186/s12911-026-03616-9 (PMC13459360; doi:10.1186/s12911-026-03616-9)
Supplement: Supplementary file 2 [file 12911_2026_3616_MOESM3_ESM.docx]

**Supplementary Table S2. Approximate model-derived SHAP transition regions for selected features in the final XGBoost model.**

| Feature | Zero-crossing (z-score) | Zero-crossing (original unit) |
| --- | --- | --- |
| ALB | -0.48011 | 35.3 g/L |
| PLT | -0.28731 | 248.4 ×10⁹/L |
| FIB | -0.39591 | 3.81 g/L |
| BMI | 0.009326 | 24.19 kg/m² |
| Na⁺ | 0.2486 | 141.2 mmol/L |
| HGB | -0.09121 | 122.5 g/L |

**Note:** Values represent approximate regions where SHAP dependence curves approached or crossed SHAP = 0 in the fitted XGBoost model. These values are descriptive of model behavior only and should not be interpreted as clinically validated thresholds, diagnostic cutoffs, treatment targets, biological tipping points, or evidence of causal effects. ALB, albumin; BMI, body mass index; FIB, fibrinogen; HGB, hemoglobin; Na⁺, sodium; PLT, platelet count; SHAP, SHapley Additive exPlanations; XGBoost, extreme gradient boosting.
