# Supplementary material for: Development, internal testing, and external validation of an interpretable machine-learning model for predicting imaging-confirmed in-hospital postoperative deep vein thrombosis in older patients with patellar fractures
Source: BMC Med Inform Decis Mak. 2026 Jun 5;26:309. doi: 10.1186/s12911-026-03616-9 (PMC13459360; doi:10.1186/s12911-026-03616-9)
Supplement: Supplementary file 4 [file 12911_2026_3616_MOESM5_ESM.docx]

**Supplementary Table S4. Distribution of thrombus location among patients with imaging-confirmed postoperative DVT in the external validation cohort.**

| DVT location | n | % among all DVT patients, n = 38 | % among classifiable cases, n = 32 |
| --- | --- | --- | --- |
| Proximal DVT | 3 | 7.9 | 9.4 |
| Distal DVT | 24 | 63.2 | 75.0 |
| Mixed proximal-distal DVT | 5 | 13.2 | 15.6 |
| Not classifiable | 6 | 15.8 | — |
| Total | 38 | 100.0 | — |

**Table note:** Proximal DVT was defined as thrombosis involving the popliteal vein or more proximal veins, including the iliac, femoral, superficial femoral, and popliteal veins. Distal DVT was defined as thrombosis confined to below-knee veins, including the tibial, peroneal, and intermuscular veins. Mixed proximal-distal DVT was defined as simultaneous involvement of both proximal and distal venous segments. Not classifiable indicated imaging-confirmed DVT cases for which detailed venous-segment information was unavailable in the retrospective records. Percentages among classifiable cases were calculated after excluding not classifiable cases. DVT, deep vein thrombosis.
