# Supplementary material for: Development, internal testing, and external validation of an interpretable machine-learning model for predicting imaging-confirmed in-hospital postoperative deep vein thrombosis in older patients with patellar fractures
Source: BMC Med Inform Decis Mak. 2026 Jun 5;26:309. doi: 10.1186/s12911-026-03616-9 (PMC13459360; doi:10.1186/s12911-026-03616-9)
Supplement: Supplementary file 5 [file 12911_2026_3616_MOESM6_ESM.docx]

**Supplementary Table S5. Detailed baseline characteristics of the internal and external validation cohorts.**

| **Variable** | **Internal cohort, n = 741** | **External validation cohort, n = 273** | **P value** |
| --- | --- | --- | --- |
| Age, years | 69.00 [65.00, 76.00] | 68.00 [65.00, 74.00] | 0.164 |
| Male sex, n (%) | 394 (53.2) | 152 (55.7) | 0.523 |
| Alcohol consumption history, n (%) | 32 (4.3) | 23 (8.4) | 0.016* |
| Smoking history, n (%) | 47 (6.3) | 27 (9.9) | 0.073 |
| Heart disease, n (%) | 140 (18.9) | 20 (7.3) | <0.001* |
| Hypertension, n (%) | 305 (41.2) | 70 (25.6) | <0.001* |
| Diabetes mellitus, n (%) | 185 (25.0) | 31 (11.4) | <0.001* |
| Cerebral infarction, n (%) | 105 (14.2) | 15 (5.5) | <0.001* |
| Open fracture, n (%) | 39 (5.3) | 49 (17.9) | <0.001* |
| ASA classification, n/N (%) |  |  | 0.178 |
| ASA I | 10/734 (1.4) | 9/271 (3.3) |  |
| ASA II | 580/734 (79.0) | 205/271 (75.6) |  |
| ASA III | 135/734 (18.4) | 52/271 (19.2) |  |
| ASA IV | 9/734 (1.2) | 5/271 (1.8) |  |
| Anesthesia type, n/N (%) |  |  | <0.001* |
| General anesthesia | 141/671 (21.0) | 27/271 (10.0) |  |
| Neuraxial anesthesia | 226/671 (33.7) | 36/271 (13.3) |  |
| Local or regional anesthesia | 97/671 (14.5) | 4/271 (1.5) |  |
| Combined anesthesia | 207/671 (30.8) | 204/271 (75.3) |  |
| Preoperative Caprini score | 10.00 [9.00, 11.00] | 10.00 [9.00, 11.00] | 0.707 |
| BMI, kg/m² | 24.10 [22.13, 26.27] | 23.00 [19.90, 27.50] | 0.011* |
| Time from injury to surgery, days | 4.00 [2.00, 6.00] | 6.00 [3.00, 10.00] | <0.001* |
| Length of hospital stay, days | 11.00 [8.00, 16.00] | 12.00 [7.00, 18.00] | 0.442 |
| Imaging-confirmed in-hospital postoperative DVT, n (%) | 137 (18.5) | 38 (13.9) | 0.106 |
| WBC, ×10⁹/L | 8.27 [6.82, 10.24] | 7.76 [6.45, 9.38] | 0.004* |
| NEU#, ×10⁹/L | 6.00 [4.57, 7.91] | 5.33 [3.95, 7.10] | <0.001* |
| NEU, % | 71.90 [65.33, 78.20] | 74.30 [66.00, 81.50] | 0.003* |
| LYM#, ×10⁹/L | 1.48 [1.16, 1.96] | 1.58 [1.25, 2.10] | 0.012* |
| LYM, % | 18.77 [13.40, 24.60] | 20.40 [13.88, 27.12] | 0.068 |
| MON#, ×10⁹/L | 0.62 [0.48, 0.78] | 0.54 [0.35, 0.75] | <0.001* |
| MON, % | 7.54 [6.20, 9.05] | 7.70 [6.30, 9.20] | 0.749 |
| EOS#, ×10⁹/L | 0.06 [0.02, 0.13] | 0.09 [0.04, 0.18] | <0.001* |
| EOS, % | 0.80 [0.28, 1.60] | 0.60 [0.12, 1.90] | 0.554 |
| BAS#, ×10⁹/L | 0.03 [0.02, 0.04] | 0.03 [0.02, 0.04] | 0.733 |
| BAS, % | 0.35 [0.20, 0.50] | 0.30 [0.20, 0.50] | 0.021* |
| IMM#, ×10⁹/L | 0.01 [0.00, 0.02] | 0.03 [0.01, 0.04] | <0.001* |
| IMM, % | 0.07 [0.00, 0.30] | 0.48 [0.22, 0.76] | <0.001* |
| RBC, ×10¹²/L | 4.09 [3.68, 4.45] | 4.53 [4.10, 4.91] | <0.001* |
| HGB, g/L | 126.90 [114.20, 139.00] | 130.00 [118.53, 143.00] | 0.060 |
| HCT, % | 37.73 [33.94, 41.26] | 40.20 [36.30, 43.20] | <0.001* |
| MCV, fL | 92.66 [89.32, 95.74] | 91.60 [87.75, 96.00] | 0.029* |
| MCH, pg | 31.25 [30.13, 32.30] | 30.30 [29.30, 31.40] | <0.001* |
| MCHC, g/L | 336.10 [330.00, 343.40] | 335.00 [327.00, 342.00] | 0.012* |
| RDW-CV, % | 13.12 [12.76, 13.51] | 12.70 [12.10, 13.45] | <0.001* |
| PLT, ×10⁹/L | 212.80 [175.00, 264.60] | 257.00 [218.15, 302.00] | <0.001* |
| MPV, fL | 8.70 [7.92, 9.43] | 10.50 [9.41, 11.02] | <0.001* |
| PDW | 16.42 [15.90, 16.91] | 14.30 [10.80, 16.31] | <0.001* |
| ALB, g/L | 39.44 [34.81, 42.30] | 41.80 [38.73, 44.31] | <0.001* |
| TP, g/L | 63.70 [59.40, 68.00] | 69.22 [65.53, 72.28] | <0.001* |
| GLOB, g/L | 23.06 [20.17, 25.90] | 26.90 [24.30, 29.20] | <0.001* |
| A/G ratio | 1.70 [1.50, 2.00] | 1.77 [1.50, 1.98] | 0.662 |
| A1/B ratio | 1.36 [1.09, 1.69] | 1.33 [1.19, 1.50] | 0.261 |
| ALT, U/L | 20.00 [13.00, 33.00] | 23.10 [16.05, 33.80] | 0.008* |
| AST, U/L | 19.00 [15.00, 29.00] | 21.50 [16.00, 29.50] | 0.190 |
| AST/ALT ratio | 1.05 [0.79, 1.40] | 1.00 [0.74, 1.36] | 0.165 |
| ALP, U/L | 62.00 [51.00, 75.00] | 83.00 [64.00, 99.97] | <0.001* |
| GGT, U/L | 22.00 [15.00, 35.00] | 24.37 [20.00, 33.64] | 0.099 |
| CHE, U/L | 7.61 [6.31, 8.92] | 8.10 [6.74, 9.53] | <0.001* |
| DBIL, μmol/L | 4.60 [3.20, 6.40] | 4.00 [2.89, 5.63] | <0.001* |
| IBIL, μmol/L | 10.57 [7.30, 14.36] | 10.10 [7.33, 13.07] | 0.322 |
| CREA, μmol/L | 65.00 [54.95, 73.80] | 64.10 [53.00, 74.60] | 0.574 |
| GLU, mmol/L | 5.76 [5.14, 6.79] | 5.41 [4.92, 6.12] | <0.001* |
| Na⁺, mmol/L | 139.96 [138.00, 142.00] | 140.07 [138.50, 142.00] | 0.381 |
| K⁺, mmol/L | 3.90 [3.63, 4.15] | 4.10 [3.89, 4.40] | <0.001* |
| Cl⁻, mmol/L | 104.70 [102.60, 107.00] | 103.95 [101.20, 105.00] | <0.001* |
| Ca, mmol/L | 2.19 [2.09, 2.28] | 2.31 [2.11, 2.40] | <0.001* |
| Mg, mmol/L | 0.89 [0.82, 0.96] | 0.89 [0.82, 0.97] | 0.243 |
| HDL-C, mmol/L | 1.15 [0.95, 1.30] | 1.27 [0.98, 1.53] | 0.362 |
| LDL-C, mmol/L | 2.56 [2.02, 3.08] | 2.33 [1.72, 3.17] | 0.003* |
| APOA1, g/L | 1.12 [0.95, 1.28] | 1.31 [1.24, 1.44] | <0.001* |
| APOB, g/L | 0.84 [0.67, 1.00] | 0.95 [0.80, 0.99] | <0.001* |
| CK, U/L | 127.00 [72.00, 309.90] | 105.07 [66.67, 192.17] | <0.001* |
| CK-MB, U/L | 12.80 [9.08, 18.00] | 13.98 [10.20, 17.98] | <0.001* |
| LDH, U/L | 187.67 [159.16, 230.00] | 188.00 [165.58, 214.21] | 0.749 |
| HBDH, U/L | 150.00 [127.20, 177.40] | 140.00 [130.00, 152.00] | 0.006* |
| PT, s | 11.80 [11.20, 12.60] | 11.90 [11.40, 12.40] | 0.303 |
| PTA, % | 93.00 [85.00, 103.00] | 95.85 [88.97, 106.25] | <0.001* |
| APTT, s | 29.60 [27.50, 32.30] | 29.00 [27.30, 31.40] | 0.013* |
| INR | 1.05 [0.99, 1.11] | 1.07 [1.01, 1.12] | 0.020* |
| TT, s | 14.40 [13.40, 15.70] | 15.90 [14.30, 17.20] | <0.001* |
| FIB, g/L | 3.26 [2.67, 3.96] | 3.31 [2.82, 3.86] | 0.551 |
| FDP | 5.80 [2.88, 13.37] | 3.00 [1.69, 3.98] | <0.001* |
| AT-III, % | 98.00 [87.00, 108.00] | 97.00 [85.00, 109.00] | 0.801 |

**Table note:** Continuous variables are presented as median [IQR], and categorical variables are presented as n (%) or n/N (%) when missing values were present. P values compare the internal and external validation cohorts using the Mann–Whitney U test for continuous variables and the chi-square test or Fisher’s exact test for categorical variables, as appropriate. *P < 0.05. For variables with missing values, percentages were calculated using the number of available observations as the denominator. Neuraxial anesthesia included spinal and/or epidural anesthesia. This detailed table summarizes candidate predictors considered before feature selection and other descriptive clinical variables used to characterize the internal and external validation cohorts.

**Abbreviations:** ASA, American Society of Anesthesiologists; BMI, body mass index; DVT, deep vein thrombosis; WBC, white blood cell count; NEU, neutrophil; LYM, lymphocyte; MON, monocyte; EOS, eosinophil; BAS, basophil; IMM, immature granulocyte; RBC, red blood cell count; HGB, hemoglobin; HCT, hematocrit; MCV, mean corpuscular volume; MCH, mean corpuscular hemoglobin; MCHC, mean corpuscular hemoglobin concentration; RDW-CV, red blood cell distribution width-coefficient of variation; PLT, platelet count; MPV, mean platelet volume; PDW, platelet distribution width; ALB, albumin; TP, total protein; GLOB, globulin; ALT, alanine aminotransferase; AST, aspartate aminotransferase; ALP, alkaline phosphatase; GGT, gamma-glutamyl transferase; CHE, cholinesterase; DBIL, direct bilirubin; IBIL, indirect bilirubin; CREA, creatinine; GLU, glucose; HDL-C, high-density lipoprotein cholesterol; LDL-C, low-density lipoprotein cholesterol; APOA1, apolipoprotein A1; APOB, apolipoprotein B; CK, creatine kinase; CK-MB, creatine kinase-MB; LDH, lactate dehydrogenase; HBDH, hydroxybutyrate dehydrogenase; PT, prothrombin time; PTA, prothrombin activity; APTT, activated partial thromboplastin time; INR, international normalized ratio; TT, thrombin time; FIB, fibrinogen; FDP, fibrin degradation product; AT-III, antithrombin III.
