# Supplementary material for: Development, internal testing, and external validation of an interpretable machine-learning model for predicting imaging-confirmed in-hospital postoperative deep vein thrombosis in older patients with patellar fractures
Source: BMC Med Inform Decis Mak. 2026 Jun 5;26:309. doi: 10.1186/s12911-026-03616-9 (PMC13459360; doi:10.1186/s12911-026-03616-9)
Supplement: Supplementary file 6 [file 12911_2026_3616_MOESM7_ESM.docx]

**Supplementary Table S6. TRIPOD+AI reporting checklist**.

| Section/topic | Item No | Checklist item | Reported in manuscript |
| --- | --- | --- | --- |
| Title | 1 | Identify the study as developing, validating, updating, or evaluating a prediction model, and indicate the modelling approach where appropriate. | Title: "Development, Internal Testing, and External Validation..." |
| Abstract | 2 | Provide a structured summary of the study objectives, design, setting, participants, predictors, outcome, model-development and validation methods, performance measures, and main findings. | Abstract: Background, Methods, Results, Conclusion |
| Introduction: Background | 3 | Explain the medical context, rationale, and intended role of the prediction model. | Introduction, paragraphs 1–3 |
| Introduction: Objectives | 4 | Specify the study objectives, including whether the study involved model development, validation, updating, or comparison with other models/tools. | Introduction, final paragraph |
| Methods: Source of data | 5 | Describe the study design, data source, setting, study dates, and whether the data were collected retrospectively or prospectively. | Methods 2.1 and 2.2 |
| Methods: Participants | 6 | Describe eligibility criteria, inclusion and exclusion criteria, and how participants were identified and selected. | Methods 2.2 |
| Methods: Prediction time point and horizon | 7 | Define the time point at which predictions are made and the time horizon over which the outcome is predicted. | Methods 2.4 |
| Methods: Outcome | 8 | Clearly define the outcome being predicted, including how and when it was measured or ascertained. | Methods 2.4 |
| Methods: Outcome clinical interpretation | 8a | Clarify whether the outcome is symptomatic, imaging-detected, proximal, distal, composite, or severity-stratified, where relevant. | Methods 2.4; Results 3.1; Discussion; Supplementary Tables S3–S4 |
| Methods: Candidate predictors | 9 | Define all candidate predictors and describe how and when they were measured. | Methods 2.3 |
| Methods: Predictor availability | 9a | Describe whether predictors are available at the intended prediction time point and whether any dynamic or post-baseline variables were excluded. | Methods 2.3 |
| Methods: Sample size | 10 | Explain the study size, number of outcome events, and any sample-size or events-per-variable considerations. | Methods 2.5 |
| Methods: Missing data | 11 | Describe missing data, exclusions based on missingness, and how missing values were handled. | Methods 2.5 |
| Methods: Statistical analysis overview | 12 | Describe all modelling steps, including preprocessing, feature selection, model development, validation, and performance assessment. | Methods 2.5–2.8 |
| Methods: Preprocessing | 12a | Describe preprocessing methods, including imputation, scaling, encoding, and whether preprocessing was fitted only on training/development data. | Methods 2.5 |
| Methods: Handling class imbalance | 12b | Describe any methods used to address class imbalance, such as SMOTE, and how data leakage was prevented. | Methods 2.5 |
| Methods: Feature selection | 12c | Describe feature-selection methods, whether they were performed within the development data only, and how selected predictors were fixed. | Methods 2.6 |
| Methods: Model development | 13 | Describe the algorithms used, model-building procedures, and hyperparameter tuning methods. | Methods 2.7 |
| Methods: Model output | 14 | Specify the model output, such as predicted probabilities or risk classifications, and describe any threshold selection. | Methods 2.7 and 2.8 |
| Methods: Validation strategy | 15 | Describe internal testing, external validation, data splitting, and whether validation data were excluded from all model-development procedures. | Methods 2.2, 2.7, and 2.8 |
| Methods: Model comparison | 16 | Describe comparison with alternative models or existing clinical risk tools. | Methods 2.7 and 2.8 |
| Methods: Performance measures | 17 | Define performance metrics used to evaluate discrimination, calibration, classification performance, prediction error, and clinical utility. | Methods 2.8 |
| Methods: Calibration | 18 | Describe how calibration was assessed, including calibration plots, intercept, slope, and Brier score where applicable. | Methods 2.8; Results 3.6 |
| Methods: Clinical utility | 19 | Describe whether and how clinical utility or decision-curve analysis was assessed, including threshold ranges and interpretation. | Methods 2.8; Results 3.6 |
| Methods: Model interpretability | 20 | Describe interpretability methods and clarify the intended interpretation of feature importance or explanation methods. | Methods 2.9 |
| Methods: Fairness and subgroup considerations | 21 | Describe whether fairness, subgroup performance, or heterogeneity was assessed, or state if not performed. | Not formally assessed; limitations discussed in Discussion and Limitations |
| Methods: Registration and protocol | 22 | Provide information on study registration or protocol availability, if applicable. | Not applicable; retrospective prediction-model study |
| Methods: Software and reproducibility | 23 | Report software, package versions, random seeds, and reproducibility information. | Methods 2.10 |
| Methods: Data and code availability | 24 | Describe availability of data and code, including restrictions due to privacy or institutional policies. | Methods 2.10; Declarations |
| Results: Participants | 25 | Report the number of participants, outcome events, and cohort flow for development, internal testing, and external validation. | Results 3.1; Figure 1 |
| Results: Baseline characteristics | 26 | Report relevant participant and predictor characteristics, including comparison between development/evaluation cohorts where applicable. | Results 3.1; Table 1; Supplementary Table S5 |
| Results: Model specification | 27 | Report the final predictors and sufficient information about the final model to support interpretation and reproducibility. | Results 3.2 and 3.3 |
| Results: Model performance | 28 | Report model performance in the internal test set and external validation cohort, including uncertainty where appropriate. | Results 3.3, 3.4, and 3.6; Tables 2–3; Figure 3; Figure 6 |
| Results: Optimism correction | 29 | Report optimism-corrected performance when used, including apparent performance, mean optimism, and corrected performance. | Results 3.3; Supplementary Table S1 |
| Results: Comparison with standard risk tools | 30 | Report comparison with existing clinical risk-assessment tools or benchmark models. | Results 3.4; Table 3 |
| Results: Outcome subtypes or additional analyses | 31 | Report clinically relevant outcome subtypes or descriptive analyses, where available. | Results 3.1; Supplementary Tables S3–S4 |
| Results: Interpretability findings | 32 | Report feature importance or explanation results while avoiding causal or threshold-based overinterpretation. | Results 3.5; Figures 4–5 |
| Discussion: Main findings | 33 | Summarize key findings with reference to study objectives and validation results. | Discussion, first paragraph |
| Discussion: Clinical interpretation | 34 | Interpret model performance in relation to intended use, clinical context, and current practice. | Discussion |
| Discussion: Limitations | 35 | Discuss limitations, including outcome definition, missing perioperative variables, confounding, sample size, overfitting, SMOTE, and interpretability limitations. | Discussion, Limitations paragraph |
| Discussion: Generalizability | 36 | Discuss transportability and generalizability across settings, including external validation and recalibration needs. | Discussion |
| Discussion: Clinical implementation | 37 | Clarify whether the model is ready for clinical implementation and what further validation or evaluation is needed. | Discussion; Conclusion |
| Other information: Ethics | 38 | Report ethics approval and consent procedures. | Methods 2.1; Declarations |
| Other information: Funding | 39 | Report funding source and role, if any. | Declarations |
| Other information: Conflicts of interest | 40 | Report competing interests. | Declarations |
| Other information: Abbreviations | 41 | Provide a list of abbreviations and ensure abbreviations are defined at first use. | Abbreviations section |

**Table note:** TRIPOD+AI, Transparent Reporting of a multivariable prediction model for Individual Prognosis Or Diagnosis + Artificial Intelligence.
